# Middle-aged and older adults’ acceptance of mobile nutrition and fitness apps: A systematic mixed studies review

**DOI:** 10.1371/journal.pone.0278879

**Published:** 2022-12-15

**Authors:** Nadja-Raphaela Baer, Julia Vietzke, Liane Schenk

**Affiliations:** Institute of Medical Sociology and Rehabilitation Science, Charité – University Medicine Berlin, Berlin, Germany; University of Ottawa, CANADA

## Abstract

**Background:**

To promote healthy aging, the support of digital mobile health tools such as mobile applications (apps) addressing a healthy diet or physical activity appears promising, particularly when initiated before entering old age. For such tools to be effective, middle-aged and older adults’ acceptance need to be studied in depth.

**Objective:**

The aim of this systematic review was to provide an integrative synthesis of the current state of research regarding the question in how far middle-aged and older adults (people aged 50 years and above) accept mobile nutrition and fitness apps to gain a deeper understanding of the influencing factors shaping this target group’s usage behaviour and needs.

**Methods:**

The review process followed the PRISMA guidelines. The databases Medline, Embase, Web of Science as well as reference lists were systematically searched. Study quality was assessed using the MMAT and AXIS appraisal tools. Data of the included studies were extracted and thereupon narratively synthesized, involving thematic analysis.

**Results:**

Of N = 8823 articles screened, n = 7 studies could be identified–five quantitative, cross-sectional ones and two qualitative studies. Overall, the synthesized findings showed a lower acceptance among middle-aged and older adults compared to younger populations, which was particularly reflected in lower usage rates and more negative attitudes towards such apps (e.g., *Perceived usefulness*, *Ease of use*). The target group’s acceptance of fitness apps was greater compared to nutrition apps. Findings on contextual factors and social determinants were inconsistent (e.g., regarding gender differences).

**Conclusion:**

While cross-study comparability was limited, the synthesized evidence underscores the importance to target mobile nutrition and fitness apps to the distinctive and heterogeneous needs of middle-aged and, particularly, of older adults. The scarcity of the existing body of knowledge highlights the need of further (longitudinal) research.

**PROSPERO protocol register number:**

CRD42020159409.

## Introduction

Age depicts a major risk factor for the development of non-communicable chronic diseases, multimorbidity, and mortality [1]. Among other lifestyle behaviors, physical inactivity as well as unfavorable dietary patterns and associated malnutrition add to the development and maintenance of such states of health [2]. Changing behavioral patterns at risk and adopting health-promoting behaviors may reduce the burden of age-related diseases. In particular, emerging evidence points at positive associations between a favorable diet and/or regular physical activity and a healthy aging process [3, 4]. Thereby, significant positive health benefits are also shown for people who only initiate regular physical activity or healthy dietary habits in later life [5]. These findings underline the great opportunities of age-specific intervention measures targeting behavioral lifestyle changes.

To effectively promote healthy aging, digital support by mHealth technologies such as mobile apps appears promising. Previous systematic reviews provide evidence for positive health effects of mHealh mobile applications, while pointing at the need for further (intervention) studies [6–10]. More specifically, fitness apps have been shown to be efficacious in motivating behavioral changes, although long-term adherence and intervention effects have been rather modest [11–14]. Similarly, the use of nutrition apps appears beneficial in supporting behavioral change towards healthy dietary patterns or weight loss, for example [8, 14–16]. Noteworthy, those investigations on fitness and/or nutrition app acceptance have so far mostly focused on younger or general populations.

While the trend towards general mHealth app usage among older people has increased, a large proportion of non-users remains [11, 17, 18]. The body of literature on the so-called *Digital Divide* between younger and older technology users indicates a generational gap regarding tendencies to (dis)engage or reject digital technologies [17, 19–21]. Accordingly, middle aged and–specifically–older people are less likely to use mHealth technologies [21, 22] and have distinct needs, attitudes as well as motivational and practical barriers towards health app usage as in comparison to younger users [18, 19, 23–25]. Yet, a comprehensive understanding of the factors that influence usage behavior is still required, to successfully promote healthy lifestyles in middle-aged and older populations by means of mobile fitness and nutrition apps [11, 17]. Hence, there is a need to understand in how far middle-aged and older people as well as adults transitioning to older age (resp. middle-aged adults) accept nutrition and fitness apps as in comparison to younger age groups.

Definitions of acceptance in the field of (health) technology research are diverse and controversially discussed [26]. This manifests itself, among others, into an incoherent, indistinct use, interpretations and operationalizations of various terminologies related to acceptance (e.g. acceptability, adoption) [26]. Alongside the existing definitional and conceptual variance, acceptance has been operationalized by a broad spectrum of dimensions, ranging from actual usage (e.g., *Frequency of use*, *Duration of use*) and user experiences (e.g., *Perceived usefulness*, *Perceived ease of use*) towards attitudes (e.g., *Desirability*, *Privacy concerns*) regarding digital tools [26–30]. Among the most established theoretical models are the (extended) Technology Acceptance Model (TAM) [30] and the Unified Theory of Acceptance and Use of Technology (UTAUT/UTAUT2) [31], which both are built on the premise that actual usage is shaped by behavioral intention.

Based on the Theory of Reasoned Action [32], the (extended) TAM focuses on the influence of *Perceived ease of use* as well as *Perceived usefulness* on the *Actual usage* of technological tools. Going beyond the initial TAM, further factors shaping technology acceptance have been added (e.g. *Fun factor* [33]). Moreover, efforts have been made to translate the TAM to the health context by including health-related constructs such as health beliefs (HITAM) [34]. While addressing similar acceptance dimensions, the UTAUT more comprehensively conceptualizes the interaction between *Usage behavior* (e.g. *Frequency of usage*) and various dimensions of *Behavioral intention* by means of four core constructs an respective operationalizations (e.g. *Performance expectancy* (e.g. operationalized by *Perceived usefulness*), *Facilitating conditions* (e.g. measured by *Perceived behavioral control*)). Accounting for various moderating factors (e.g. age, gender), the UTAUT 2 has added three further constructs, namely *Hedonic motivation*, *Price value* and *Experience and habit* (e.g. referring to automatized usage). Exceeding general technology acceptance, both models have been applied to various technologies, such as digital mHealth tools. However, the applicability of the UTAUT 2 model to mHealth acceptance has recently been contested [35]. Moreover, in applying and extending such existing acceptance models, studies have primarily addressed mHealth apps in general and targeted broad population groups as well as relatively young age groups [35–41].

So far, systematic reviews particularly focusing on nutrition and/or fitness app acceptance have also addressed broader and younger target groups [42–44]. For instance, a systematic review dealing with nutrition app acceptance has provided a conceptual framework on a great number and diversity of barriers to and facilitators for app usage among their target population of adolescents and adults. Based on their findings, the authors call for a more pronounced “tailoring [of] nutrition apps to the needs of specific user groups” [42]. To do so, knowledge on specific usage groups–such as adults of middle and older age–seems vital. With respect to the current body of knowledge, a systematic examination of the scientific evidence regarding nutrition and fitness app acceptance among these particular age groups is yet lacking. An integration of the present body of knowledge is hence needed to guide future research and practice–such as app developers and actions for public health.

Against this background, this systematic mixed-studies review addresses the following research questions: To what extent do middle-aged and older adults accept mobile nutrition and fitness apps and what influencing factors and social determinants shape their acceptance? In how far does the acceptance among adults aged 50 years and older differ compared to younger population groups? Exploring these research questions, this systematic review aimed to synthesize findings of qualitative, quantitative as well as mixed methods studies as comprehensive as possible, thereby covering the existent evidence along the entire spectrum of acceptance dimensions that inductively emerge throughout the screening process (i.e., for instance, going beyond the (extended) TAM and UTAUT/2). The present article provides an integrative overview of the existing evidence, points at research gaps and discusses directions for future research.

## Materials and methods

This systematic review complied with the PRISMA-guidelines [45, 46] (see S1 Appendix).

### Review protocol

A review protocol was registered with the PROSPERO International Prospective Register of Systematic Reviews (ID: CRD42020159409), where the review progress and deviations from the original protocol were documented with a last update in September 2022.

### Eligibility criteria

Studies were eligible for inclusion if they investigated middle-aged and older adults’ acceptance of nutrition and/or fitness apps. More specifically, research on any dimension(s) of app acceptance–such as frequency of use or perceived usefulness–was eligible. Only those studies were included, which addressed *general* nutrition and/or fitness apps. Hence, studies focusing on nutrition or fitness apps specific for disease management or treatment were excluded (e.g., diabetic apps).

With respect to the target group, studies with a sample involving community-dwelling participants aged 50 years and older were considered for inclusion. Older age is generally defined as starting with 60 to 65 years in industrialized countries [11]. To account for adults of middle-age–i.e. those transitioning to older age–as well as for potential socio-cultural differences of conceptualizations of “older age” [47], we set the criterion for our target group at 50 years and above. Studies reporting age effects (or the lack thereof) were eligible, including both studies that treated age as a continuous variable and those that explicitly addressed different age groups. Therefore, studies which did not exclusively target middle-aged and/or older adults but included older age groups were also eligible for inclusion. Since our study aimed to explore app acceptance in general populations, investigations on samples of institutionalized individuals or patient collectives were not eligible for inclusion. Only original articles published in English, German or French language were considered. Intervention and feasibility studies as well as studies on clinical research participants were likewise excluded, to facilitate the synthesis of findings generated under (relatively) “real-world” conditions. Grey literature or non-original articles such as conference abstracts was not sought. Table 1 provides a summary of the a priori defined inclusion and exclusion criteria in accordance with the PICOS-scheme.

**Table 1:** Inclusion and exclusion criteria according to the PICOS criteria^1^.

PICOS component	Inclusion criteria	Exclusion criteria
**Participants**		
	Samples including adults aged ≥ 50 yrs. Community-dwelling adults	Studies exclusively targeting age groups < 50 yrs. Institutionalized adults (e.g., living in a care facility) Studies focusing on patient collectives or clinical research participants
**Intervention/Context**		
	Studies researching older adults’ acceptance of general nutrition and/or fitness apps compared to younger adults	Studies with a focus on disease management or treatment apps were excluded (e.g., nutrition app for managing diabetes) .
**Outcomes**		
	Any dimension(s) of nutrition and/or fitness app acceptance defined by the studies’ authors (e.g., frequency of usage, perceived usefulness) Age effects, i.e., age-group-specific differences in nutrition and/or app acceptance	Studies focusing on general health apps without presenting specific results for either nutrition or/and fitness apps Studies not reporting any age effects (or the lack thereof), i.e., age-group-specific results regarding nutrition and/or fitness app acceptance
**Study design**		
	Original articles Qualitative studies Quantitative studies Mixed methods studies	Intervention studies, feasibility studies (e.g., evaluating the effectiveness of nutrition and/or fitness apps) Non-original articles (e.g., conference abstracts, book chapters) Literature reviews Grey literature

***Note***.

^**1**^PICOS = Population, Intervention/ Context, Outcomes, and Study Type; yrs. = years

### Search strategy and selection process

Following a pilot search, a systematic search of the databases Medline, Embase and Web of Science was conducted from June to August 2022. The same search strategy was used for each database, with adjusted search terms according to the requirements of the respective database (see S2 Appendix). The final search strategy was developed by two authors and adjusted after consultation by a professional librarian.

Titles of all search results were checked for relevance with respect to the research question. Potentially relevant articles were imported into a bibliographic management software program (Endnote 20), to remove duplicates and to keep track of the selection process. Publications with relevant titles were selected for further consideration. After abstract screening, full texts were read and selected for inclusion if they met the inclusion criteria. Subsequently, reference lists of the included articles were scanned by hand search and passed through the same systematic search process as the original one. The selection process was conducted by two researchers. Ambiguities were discussed and consensus reached in all cases.

### Data extraction

Data of the included studies were systematically extracted and collected in a piloted data extraction sheet (see S3 Appendix). Data were obtained only when deemed relevant to the purpose of this review; that is, information on hypotheses or outcomes, for example, was not obtained if it did not relate to the acceptance of nutrition and/or fitness apps in our target population (e.g., in some cases, hypotheses and results were presented on general of mHealth app usage). Main categories were *General information* (e.g., title, journal, geographical context), *Research foci* (e.g., research aims, hypotheses), *Study design and methods* (e.g., sampling, data gathering methods, analysis methods), *Sample and participant characteristics* (e.g., sample size, age, mobile phone ownership, app usage, *Study outcomes* (usage of nutrition or fitness apps, attitudes towards nutrition or fitness apps). Discussions of the included studies were not extracted to avoid potential bias in the synthesis by the interpretations of the primary studies’ authors. In two cases, study authors were approached for further details because information provided was ambiguous. Data extraction was carried out by the first author and subsequently double-checked by a second researcher.

### Quality appraisal

To evaluate the methodological quality of the included studies, we first used the validated MMAT tool, which facilitates a critical appraisal of qualitative, quantitative as well as mixed-methods studies by means of a synchronized criteria catalogue [46]. Additionally, the MMAT criteria are relatively broad and leave room for individual interpretation, rendering them prone to bias. In a second step, were therefore additionally assessed the quantitative studies by means of the AXIS tool, which enables a more standardized in-depth assessment of the quality domains also proposed by the MMAT. Two authors independently completed the appraisal checklists and critically assessed the overall results. To synchronize and validate the resulting appraisals of the quantitative studies, both authors checked the checklists for congruence. In case of disagreement, consensus was reached by discussion (see S4 Appendix for the reconciled checklists). As suggested by the developers of the MMAT as well as the AXIS tools [46, 48], no overall score was derived from the two sets of criteria ratings. Instead, a comprehensive presentation and discussion of the ratings provides details on the outcomes of each study’s quality appraisal. The risk of bias across studies was evaluated within the research team. The strength of the cumulative evidence of the included studies is reflected in the discussion section.

### Data synthesis

Quantitative as well as qualitative studies were included that were based on various research designs and showed heterogeneous study foci and characteristics, so that a quantifying synthesis tool (e.g., meta-analysis) was not considered appropriate. Therefore, a narrative synthesis design [49] was used, which comprised three major steps: (1) organization of the included studies; (2) analysis of the findings within studies, i.e. a narrative description of each study’s findings as well as of the methodological quality; and (3) a cross-study synthesis aiming at exploring interconnections and (in-)congruencies, thereby providing “an overall summary of the study findings taking into account of variations […] that may affect the generalizability of the results” [49]. Steps 2 and 3 were carried out by means of thematic analysis. In doing so, (sub-)themes related to older people’s acceptance of fitness and nutrition apps were retrieved inductively (see Tables 3 and 4). Thereafter, the identified (sub-)themes were clustered. Each step of the synthesis process was undergone by two authors.

## Results

### Search results

**Fig 1:**
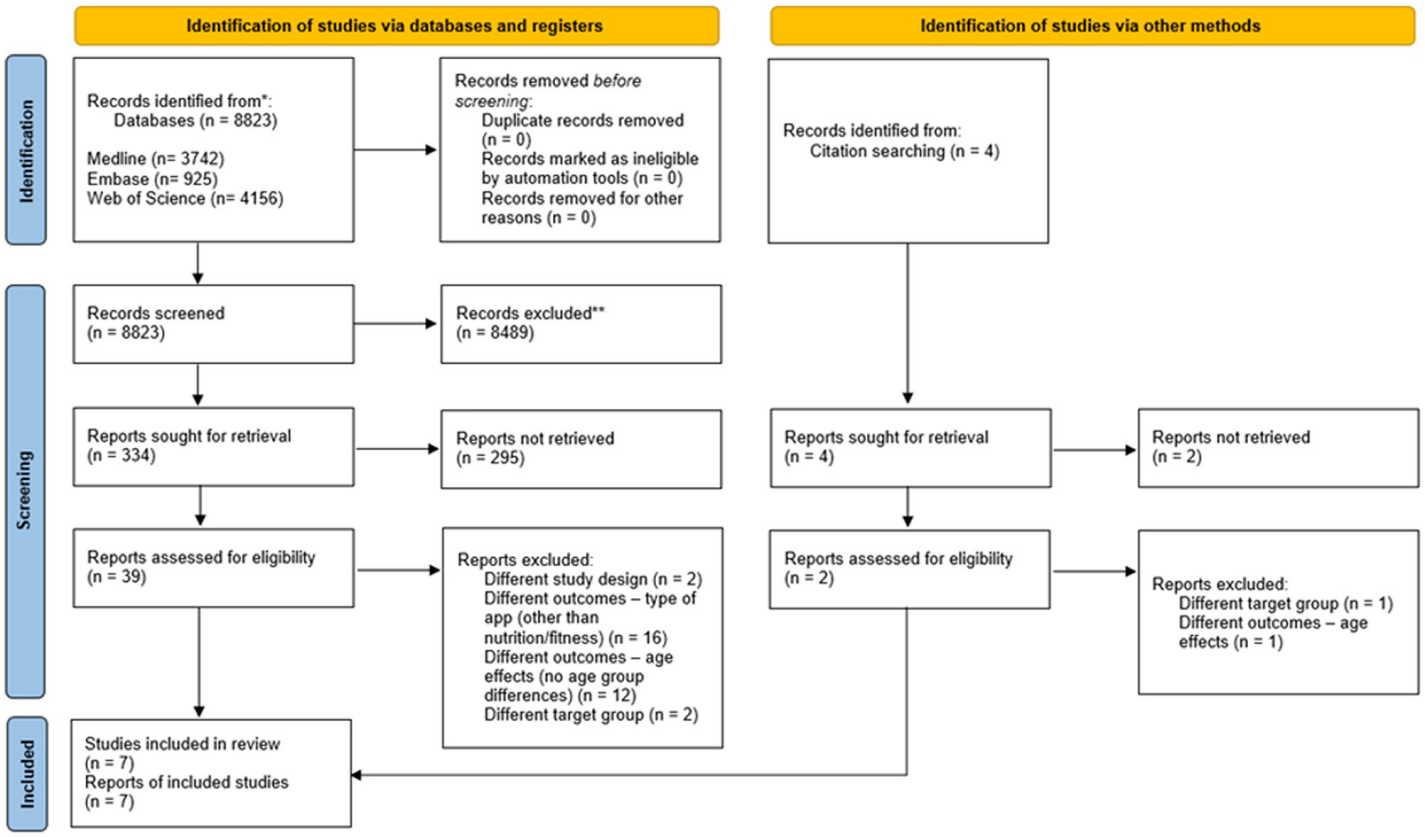
PRISMA flow diagram.

In total, the systematic search yielded N = 8.823 results, of which n = 338 publications were identified as relevant by means of title screening. The majority of studies were excluded due to different study outcomes (e.g. different type of apps). Abstract screening resulted in n = 41 publications, which were selected for full-text screening. Subsequently, n = 6 articles were identified for inclusion and one additional article was identified by hand search. In total, n = 7 articles were included for synthesis. Fig 1 illustrates the selection process according to the PRISMA guidelines [50].

### Study and sample characteristics

The identified seven articles were published between 2016 and 2019. Except for one US American study [51], all studies were conducted in Europe: in Germany [52, 53], the Netherlands [54, 55], Austria [56] and Switzerland [57]. One study was a longitudinal qualitative one [55], while the other investigations were based on quantitative, cross-sectional research designs using questionnaire surveys. The included studies varied considerably in their research foci and the extent to which they addressed older adults’ acceptance towards fitness and/or nutrition apps. All studies but one [57] addressed both nutrition as well as fitness apps, four of which [51, 54–56] dealt with general mHealth technologies and apps including those on nutritional habits and physical activity. The study by König et al. [52] was the only one specifically on nutrition and fitness app acceptance and Seifert et al. [57] exclusively targeted fitness apps.

Moreover, the included studies differed in terms of their target populations, particularly regarding age and technology use (see Table 1). Total sample sizes, i.e., including older adults as well as all age groups covered by the quantitative studies, varied substantially–with N = 562 study participants having constituted the smallest [56] and N = 4974 the largest sample [51]. In the qualitative study by Cabrita et al. [55], a total of N = 12 older adults participated in the first semi-structured interview, and N = 11 in the case study and subsequent second interview. Wichmann et al. [53] conducted three focus groups discussions with a total of n = 15 participants. One focus group with n = 5 participants included fitness app users, while the two others (n = 4, n = 6) involved non-users only. Three studies only [53, 55, 57] addressed the review question specifically regarding the age group of older adults, while the other studies addressed adults in general, including middle-aged and older ones. Therefore, mean ages differed greatly, ranging from 36.9 years (r = +/-1.2) [56] up to 69 years (r = 65–78) [55]. However, samples were a priori stratified by age in two studies: Seifert et al. [57] examined age group differences among older adults by analyzing three subgroups (n_50–64 yrs. = 522; n_65–79 yrs. = 358; n_80+yrs. = 133). Moreover, in the study by Naszay et al. [56], the two age groups ‘digital natives’ (n<35 yrs. = 305) and ‘digital immigrants’ (n>35 yrs. = 257) were contrasted. These two digital age groups did not differ with respect to gender, health profession or place of living. Yet, ‘digital immigrants’ were more likely to have graduated from tertiary education than ‘digital natives’ (*P*< .001).

With regard to gender, the samples of Seifert et al. [57] and Bol et al. [54] were almost equally distributed, with a slightly higher proportion of female participants. The other studies had a higher number of female participants in their samples, with 64% women in König et al. [52], 58% women in Mackert et al. [51] and 59% female participants in Naszay et al. [56]. Among the participants in Cabrita et al. [55], seven were female and five male. In Wichmann et al. [53], gender distributions were unequal across the focus groups: all participants of the focus group with fitness app users were male, one group of non-users was entirely female (n = 4) and the other one consisted of four female and two male interviewees. See Table 2 for more details (further information is presented in the data extraction sheet, S3 Appendix).

**Table 2:** Overview study and sample characteristics.

Authors (year of publication)	Place	Target population	Study focus (type of app)	Study design	Sampling; (rr); representativity	Sample size (N(m/f))	Mean age +/- SD; (r) in yrs.; age groups (n)	Data gathering methods
**Bol et al.** [54] (2018)									
	The Netherlands	smart device owners; Dutch adults (aged >18 yrs.)	general health apps, including nutrition and fitness apps	quantitative, cross-sectional study	sample drawn from a panel based on a representative sample of the Dutch population (CentERdata’s LISSPANEL); rr not provided	1079 (495/584)	50.32 +/- 16.35; (r = 18–89)	standardized questionnaire; online survey
**Cabrita et al.** [55] (2019)									
	The Netherlands	general population; Dutch community-dwelling older adults (target age group not specified)	general health apps, including nutrition and fitness apps	qualitative (case) study	sample drawn from 1. local information markets to promote healthy behaviors in the region of Overijssel (NL), 2. information sessions given to participants in the European Project PERSSILLA	12	69; (r = 65–78)	qualitative semi-structured interviews (pre and post app use within case study; *Note*: for the purpose of this review, only results prior to app exposure are reported)
**Wichmann et al.** [53] (2019)									
	Germany	German fitness app users and non-users (aged >50 yrs.)	fitness apps	qualitative, cross-sectional study	participants recruited via the associated online survey of the mixed methods study; offline: flyers, gatekeepers of sport clubs and other initiatives; online: advertisements	N = 15 (7/8) individuals; 3 focus groups: 1. app users: n = 5, n_m = 5, 2. non-users. n = 4, n_f = 4, 3. non-users. n = 6, n_f = 4	61.3 +/- 8.7; 3 focus groups: 1. fitness app users: 63.0 +/- 4.5, 2. non-users: 68.8 +/- 9.8, 3. non-users: 55.0 +/- 6.6	qualitative focus group discussions
**König et al.** [52] (2018)									
	Germany	general population; German adults (aged >18 yrs.)	nutrition and fitness apps	quantitative, cross-sectional study	sample drawn from a local longitudinal cohort study (Konstanz Life Study); rr not provided	1215 (432/783)	41.11 +/- 17.56	standardized questionnaire; paper-pencil survey
**Naszay et al.** [56] (2018)									
	Austria	Internet users; Swiss adults (target age group not specified)	general health apps, including nutrition and fitness apps	quantitative, cross-sectional study	four-phase snowball-sampling (offline (e.g. health-related professional associations) & online (e.g. health forums, Facebook); rr not provided	562 (231/331)	36.9 +/- 1.2; n<35 yrs. = 305 n≥35 yrs. = 257	Self-validated standardized questionnaire (validated by pilot test with N = 20 health professionals); online survey
**Mackert et al.** [51] (2016)									
	USA	patients (not specified), American adults (target age group not specified)	general health apps, including nutrition and fitness apps	quantitative, cross-sectional study	sample drawn from an invitation-only research panel (not specified);rr not provided; representative for the USA demographic composition regarding gender, age, ethnicity, socioeconomic status	4974 (2102/2872)	43.5 +/- 16.7	standardized questionnaire; online survey
**Seifert et al.** [57] (2017)									
	Switzerland	general population; Swiss older adults (aged >50 yrs.)	fitness apps	quantitative, cross-sectional study	simple random sample drawn from commercial AZ-Direct database (based on public phone book); rr = 18%; sample representative for age, gender, education, language region	1013 (475/538)	65.3; SD not reported (r = 50–80+); n_50–64 = 522 n_65–79 = 358 n_≥80 = 133	standardized questionnaire; computer assisted telephone interview

***Note*.** yrs. = years; rr = response rate; m = male, f = female; SD = standard deviation; r = range

### Quality appraisal

#### MMAT criteria

All of the included studies met the two screening questions, since clear research questions were stated, and the data collected were adequate in addressing these. The qualitative study by Cabrita et al. [55] fulfilled the further criteria whether 1) the qualitative approach and 2) the methods of data collection were adequate to answer the research question. Yet, two quality criteria were not met by this study: the interpretation of the results appeared to lack sufficient substantiation by the data and the data sources, analysis and interpretation of the results did not appear coherent. Moreover, in how far the findings were adequately derived from the data could not surely be answered (“can’t tell”) due to missing information. This was also the case for the qualitative study by Wichmann et al. [53], which, apart from that, met all MMAT criteria.

Of the quantitative studies, one study met all quality criteria [54]. For three studies [52, 56, 57], all criteria were met, except whether the risk of non-response bias was low. Here, rather than a high response bias observed, no information was provided by any of these studies. For the study by Mackert et al. [51], two further quality criteria had to be answered with “can’t tell”: Whether the sampling strategy was relevant to address the research question and whether the sample was representative for the target population.

#### AXIS criteria

The AXIS quality criteria regarding the introduction were met by all the quantitative studies. Moreover, the study designs were appropriate for accomplishing the studies’ aims. While the target populations were clearly defined in the majority of studies, two did not provide precise definitions on the selected age group “adults” [51, 56]. Moreover, two publications lacked reasoning for their sample sizes [51, 57]. None of the included studies reported on their selection process in a way that allows inferences to be drawn about the likelihood of a representative sample. However, Mackert et al. [51] and Seifert et al. [57] referred to their samples as being representative concerning specific demographic characteristics. The majority of studies [52, 54, 56] met further quality criteria regarding the methods used and presented, e.g., a proper sample frame selection, utilization of valid instruments to measure risk factors and outcome variables, as well as a clear description of the statistical methods. One study met all such criteria, except for the use of trialed data gathering methods [57]. Another study did not meet the AXIS quality criteria in terms of the sample frame selection and an adequate description of the statistical methods [51]. None of the studies included information on non-responders and one study only mentioned a response rate [57].

Most quality criteria concerned with the result sections were met by all studies, including, for instance, an adequate, internally consistent data description. Overall, the discussions also met the AXIS criteria. Additionally, no conflicts of interest or conflicting funding sources were declared in any of the studies. Ethical approval was obtained from the responsible ethics committees or institutional review boards in all studies, except for the study by Seifert et al. [57] who highlighted Swiss legal standards, based on which no ethical approval was required. Several criteria could not be assessed unambiguously because necessary information was not reported. For example, this was the case in all studies regarding whether measurements were taken to address non-responders. For more details, see S4 Appendix.

### Nutrition and fitness app acceptance: (Sub-) themes resulting from the narrative synthesis

Resulting from the thematic analysis, two overarching themes emerged concerning the acceptance of nutrition apps and fitness apps from all included studies: (1) *Usage* and (2) *Attitudes*. A third overarching theme–*Wishes and expectations–*was solely derived from the qualitative studies [53, 55]. The included studies differed in their conceptualizations of app acceptance: While all studies considered both actual app usage as a dimension of acceptance as well as various attitudes toward apps, they varied regarding the subthemes of the latter. Subsequently, the identified (sub-)themes and related findings are presented. Table 3 shows the (sub-)themes generated based on the quantitative studies and indicates the extent to which the different dimensions of acceptance are addressed by each of the studies. The (sub-)themes that emerged from the synthesis (thematic analysis) of the qualitative studies by Cabrita et al. [55] and Wichmann et al. [53] are summarized in Tables 4 and 5.

**Table 3:** Age group-specific acceptance of nutrition and fitness apps (middle-aged/older adults vs. younger adults).

	Acceptance of nutrition apps (n)					Acceptance of fitness apps (f)					
	I. (n) usage	II. (n)				I. (f) usage	II. (f)				
		attitudes					attitudes				
		II.I (n) perceived usefulness	II.II (n) ease of use	II.III (n) interest/ desirability	II.IV (n) privacy concern		II.I (f) perceived usefulness	II.II (f) ease of use	II.III (f) interest/ desirability	II.IV(f) privacy concern	I.V(f) reason for usage
[54]	**X**	**−**	**−**	**−**	**X**	↓*	**−**	**−**	**−**	**X**	**−**
[56]	**X**	**−**	**−**	↓*	**−**	↓*	**−**	**−**	↓*	**−**	**−**
[51]	**X**	↓*	↓*	**−**	↑*	**−**	↓*	↓*	**−**	↑*	**−**
[52]	↓*	**−**	**−**	**−**	**−**	↓*	**−**	**−**	**−**	**−**	**−**
[57]	**−**	**−**	**−**	**−**	**−**	↓*	**−**	**−**	**−**	**−**	documentation for physician ↑*
											PA tracking ↓*

**−** not examined or reported; **X** no (significant) age groups differences examined /found; **↓** lower likelihood for older adults, **↑** higher likelihood for older adults; *significant effect(s); PA = physical activity. *Note*: age effects illustrated here are based on different definitions and operationalizations of middle aged and older age (see Table 2).

**Table 4:** Fitness and nutrition app acceptance investigated by the qualitative study by Cabrita et al. [55].

Theme	Subtheme(s)			Specifications
**app usage**	**fitness app**			no current usage
	**nutrition app**			no current usage
				no future usage (no need, self-perceived high activity level (n = 2))
**attitudes towards app** (pre usage)	**fitness app**	**interest / desirability**	**barriers / reluctance to usage**	
			**general**	*to the beginning of the interview*: overall reluctance / rejection (n = 12) *during the course of the interview:* hypothetical usage (“*maybe*”) (n = 6), still rejecting (n = 6)
			**perceived usefulness**	no benefit seen, preference to rely on own bodily feelings to assess activity level (n = 5)
			**expected behavior change**	usage leads to decreased attention to body signals (n = 1)
			**privacy concerns**	none mentioned
	**nutrition app**	**interest / desirability**	**barriers / reluctance to usage**	
			**general**	vague, hypothetical interest after specific suggestions by interviewers (“*maybe that could be something*” answered) (n = “mostly answered”)
			**perceived usefulness**	self-monitoring dietary practices established, no need of technological support (n = “some participants”) digital monitoring too time-consuming (n = “others”) preference to talk to someone (n = 1)
			**privacy concerns**	none mentioned
**wishes and expectations towards apps**	**fitness app**	**wishes and expectations**	**monitoring functions**	overview daily physical activity regarding intensity, number of steps (n = 6) overview daily distance walked and biked (n = 2) overview calories burnt (n = 2) distinction between activities performed indoors/outdoors (n = 1)
			**motivating features**	personalized coaching of daily physical activity goals, tailored to health status, age and gender (n = 3) setting of own activity goals (n = 1) gamified coaching system, e.g., collecting points (n = 1)
			**social exchange features**	comparison to activity of peers (“most participants”)
		**expectations**	**positive**	expected behavioral adaptation according to feedback (n = “most participants”)
			**negative**	distraction by digital monitoring (“*attention theft*”) (n = 1) overview of time spent inactive too confronting (n = 1)
	**nutrition app**	**wishes**	**monitoring functions**	caloric and /or nutritional intake (n = 4)
			**motivating features**	healthy recipes tailored to medical background and needs (n = 4)
			**social exchange features**	sharing of nutritional knowledge with peers (n = 1)

*Note*: Themes, subthemes and specifications presented here are a result of the review synthesis process resp. thematic analysis, i.e., this table depicts an abstraction resp. deduction of the primary study [55] results and only involves study results addressing the review questions.

**Table 5:** Fitness app acceptance investigated by the qualitative study by Wichmann et al. [53].

Theme	Subtheme(s)		Specifications
**app usage**	**readiness to future fitness app usage**		greater among fitness app users (FG2/3) compared to non-users (FG1)
**attitudes towards fitness app usage**	**general attitudes**		more positive among fitness app users (FG2/3) compared to non-users (FG1)
	**barriers** (across all FG)	**perceived ease of use**	lack of easy handling, simplicity, practicability of usage manual data entry permanent carrying of mobile phone (preference for wristband)
		**privacy concerns**	potential data use by health insurance companies potential data use for commercial purposes exception: if app positively promoted by others and willingness to usage high, then the reluctance to disclose data would decrease
		**technologization skepticism**	unwillingness to be “dictated” by app, demand for autonomous decision-making regarding intensity of movement independently fitness apps generally disadvantageous as ability of self-perception diminishes due to technologization
	**facilitators** (across all FG)	**perceived usefulness**	useful for initiation of PA (“majority of participants”); conditions:1. if fixed aims are set, 2. if self-discipline and motivation existent motivation for the initiation of increasing fitness levels helpful in promoting a general healthy lifestyle support for creating diverse and intensive sport programs
**wishes towards fitness apps**	**general functioning** (across all FG)		maximum ease/simplicity of use
	**specific features** (across all FG)	**monitoring function**	automatic, non-manual sensory activity tracking activity tracking/monitoring
		**feedback function**	feedback on self-control by means of reminders feedback by means of social comparisons (e.g. challenges, comparison of success with others)

FG = focus group. FG1 = focus group with fitness app users, FG2 and FG3 = focus groups with non-users. PA = physical activity. *Note*: Themes, subthemes and specifications presented here are a result of the review synthesis process resp. thematic analysis, i.e., this table depicts an abstraction resp. deduction of the primary study results and only involves study results [53] addressing the review questions.

#### Nutrition and fitness app usage

All of the included studies investigated middle-aged and/or older adults’ usage (rates) of nutrition and/or fitness apps. In the quantitative studies, operationalizations of app usage, reference groups or subsamples included in the analyses as well as the extent to which usage frequencies and associated correlates were examined varied considerably. For instance, while two studies only provided frequency distributions for various age groups [56, 57], the others [51, 52, 54] did not stratify for age. Accounting for such conceptual and methodological divergences, this chapter provides an integrated overview of the synthesized results regarding the acceptance dimension actual *usage*.

#### Nutrition app usage

In the study by Seifert et al. [57], 13% of smartphone and/or tablet users aged 50 years and older (n = 719) used nutrition apps. Similarly, for their total sample of smart-device owners (N = 1079), Bol et al. [54] showed that nutrition apps were used by 27.7% of the subgroup of mobile health app users. Those results concern the study’s total sample (mean age, SD = 50.32 yrs., +/- 16.35, r = 18.89) and were not provided specifically for older adults. In Mackert et al. [51], 33.98% of their sample of adults aged 43.5 years (N = 4974, SD = +/-16.7) indicated having ever used a nutrition app.

Moreover, Naszay et al. [56] found that–in their sample of Austrian adults (N = 562; mean age, SD = 36.9 yrs., +/-1.2)–apps monitoring nutritional habits (e.g., calorie intake) were the second most frequently used type of health apps, following fitness apps (13.5%, 95% CI 10.9–16.4). This also applied for the two age subgroups *digital natives* (<35 yrs.; 15.1%, n = 395) and *digital immigrants* (>35 yrs.; 11.7%, n = 257). Comparative analyses of those two strata did not show any significant differences [56]. In neither of these studies, frequency distributions of nutrition app usage were stratified specifically for the age group of adults aged 50 years and older. Yet, further social determinants were investigated: Analyses of demographic factors associated with nutrition app usage conducted by Bol et al. [54] did not show any significant age differences. However, gender differences were found as men were less likely to use nutrition apps compared to women (OR = 0.28; 95% CI 0.16–0.50).

König et al. [52] showed that 7% of their sample of German adults (N = 1215; mean age, SD = 41.11 yrs., +/-17.56)–including general health app as well as non-users–had ever installed a nutrition app. Among all participants who owned a mobile device (n = 1051), 76.69% had never installed a nutrition app, while 15.13% reported having previously installed a nutrition app and 8.18% indicated having currently done so. Regarding usage frequency among all app users (n = 86), 37.65% used a nutrition app at least once a day. The authors further showed that older study participants (not specified) were significantly more likely to be “unengaged” with nutrition apps than younger ones who faced the behavior stages “decided to act”, “acting” and “disengaged” more often (F_4252.00_ = 16.85, *P* < .001, w2 = .06) (König et al. [52]). This study also revealed that, compared to men, women were significantly more often categorized as “unengaged” (n_female_ = 312/643, n_male_ = 221/374; *P* = .001) and “disengaged” (n_female_ = 103/643, n_male_ = 37/374; *P* = .006).

While in the qualitative study by Cabrita et al. [55], none of the participants had used nutrition apps prior to the study, one of the focus groups in Wichmann et al. [53] consisted of fitness app users (n = 5) and participants of the other two groups (n = 4, n = 6) had never used any fitness app before.

#### Fitness app usage

In the study by Wichmann et al. [53], participants of one focus group (n = 5, 63.0 yrs., 4.5 SD, all male) had previously used fitness apps, whereas the other two groups consisted of non-users only. Similar to nutrition app usage, none of the twelve participants interviewed by Cabrita et al. [55] had previously used fitness apps. In contrast, among the considerably younger sample of smart-device owners in Bol et al. [54] (mean age, SD = 50.32 yrs., +/-16.35; r = 16.35), 52.3% of mobile health app users (n = 310/1079) used fitness apps, whereby men were more likely to use fitness apps than women (OR = 2.30, 95%CI = 1.42–3.74). In addition, the authors found older study participants (not specified) significantly less likely to use fitness apps compared to younger ones (OR = 0.97; 95%CI 0.96–0.99). In Mackert et al. [51], 27.64% of the participants (N = 4974; mean age, SD = 43.5 yrs., +/- 16.7) had ever used a fitness app [51].

Evidence by König et al. [52] holds that approximately 21% of their total sample of German adults (N = 1215; mean age, SD = 41.11 yrs., +/- 17.56) had used fitness apps. Self-reported installation rates demonstrated that 52.33% of the subsample of mobile device owners (n = 1051) had never installed a fitness app. Contrary to nutrition apps, participants used fitness apps three times as often (n = 86/255) (not stratified for age). At the same time, most fitness app users reported using their apps several times per week (36.7%, n = 94/255). König et al. [52] examined sociodemographic correlates of the different behavioral adoption stages suggested by the Precaution Adaption Model (PAPM). Regarding fitness app usage, significant age differences were identified between the five behavioral adoption stages (F_4252.00_ = 22.38, *P* < .001, w^2^ = .08), with older people having been significantly more likely to be classified as “unengaged”, compared to the stages “acting”, “decided to act” and “disengaged”. In contrast to their findings on nutrition apps, König et al. [52] did not find any significant gender differences. In the Austrian study by Naszay et al. [56], 19.6% of the total sample of Internet users (N = 562; mean age, SD = 36.9 yrs., +/- 1.2) had currently used exercise apps, of which 23.9% were *digital natives* and 14.4% *digital immigrants* (95% CI 16.4–22.8, *P* = .005) [56].

Seifert et al. [57] examined fitness app usage in a general population of Swiss adults aged over 50 years. Findings demonstrated that 15% of the participants (N = 1013; mean age, SD = 65.3 yrs., SD not reported) used at least one app for activity tracking, half of whom (51%) used such an app daily. Subgroup analyses revealed that the group of the 50–64 years old participants used smartphone and/or tablet apps for physical activity tracking significantly more often than those aged between 56–79 years and above 80 years (Cramer’s V = 0.10, *P* = .04). Moreover, fitness app users were more likely to be male than female (Cramer’s V = .10, *P* = .006) [57].

#### Attitudes towards nutrition and fitness apps

*Attitudes towards nutrition and fitness apps* emerged as a central theme that unfolded in three major subthemes: Based on the findings of three quantitative [51, 56, 57] and both qualitative studies [53, 55], *Perceived usefulness*, *Ease of use* and *Interest in usage* could be derived as central *Facilitators* of usage, i.e. the initiation and/or maintenance of fitness and/or nutrition app use. Closely related, the second subtheme *Wishes and Expectations* was derived from the qualitative studies. Third, two major *Barriers*–*Lacking ease of use* and *Privacy concerns*–were dealt with by four studies [51, 53–55]Subsequently, the synthesized evidence is presented for both, nutrition and fitness apps.

#### Facilitators: Perceived usefulness, ease of use and interest in usage

According to study by Mackert et al. [51], older study participants (not specified) perceived nutrition apps (ß = -0.54, *P* < .001) and fitness apps (ß = -.106, *P* < .001) significantly less useful compared to younger ones. Besides, older age was associated with a significantly lower ease of use regarding fitness and nutrition apps (ß = -.204, *P* < .001; ß = -.145, *P* < .001).

In Naszay et al. [56], *digital immigrants* showed a significantly lower interest in fitness (*P* = .001) and nutrition app use (*P* = .001) as in comparison to *digital natives*. Within this younger age group of fitness app users (n = 305, <35 yrs.), 60% showed “low interest”, whereas 74.7% of the older age group (n = 257, >35 yrs.) reported little interest. These distributions were similar for the desirability of nutrition apps: While approximately half (51.8%) of the *digital natives* subsample (n = 305) was hardly interested in nutrition apps, this applied to 73.9% of the *digital immigrants* (n = 257) [56].

Examining *Perceived usefulness* resp. *Reasons for fitness app* usage, Seifert et al. [57] found the majority (65.8%) of their subsample of participants aged 50 years and older, who digitally tracked their physical activity (n = 208), to use such apps “to track daily physical activity” [57]. This function was significantly more often selected by the younger age groups (50–64 yrs., 65–79 yrs.) than by older users (>80 yrs.) (Cramer’s V = .20, *P* = .02). Moreover, 58.9% of this subsample (n = 208) indicated using an app to track physical activity as a motivational tool “to remain healthy” [57]. 21.5% reported exchanging personal data on physical activity with friends by means of fitness apps, while 17.2% used an app to digitally document such data for their physicians. The latter was significantly more often reported by older study participants (>65 yrs., n = 73) compared to younger ones (50–64 yrs., n = 135) (Cramer’s V = .30, *P* < .001) [57].

The subthemes *Interest in usage* and *Perceived usefulness* also emerged from the qualitative study by Cabrita et al. [55] that focused on older adults (mean age = 69yrs., r = 65–78), who had never used mobile apps before. The extent to which participants were interested in nutrition and/or fitness apps and perceived them as useful changed not only during the course of the study, but also during the first interview: Initially, the authors found a relatively pronounced rejection of fitness (n = 12) as well as nutrition (n = 6) apps. However, during the first interview, in which further information on potential functions of such apps were provided, half of the participants changed their attitudes somewhat and could imagine using a fitness app, while the other ones remained rejecting. Similarly, most participants showed a hypothetical, rather vague (“maybe”) interest in nutrition apps [55].

In Wichmann et al. [53], focus group participants who already had used fitness apps showed a greater readiness for future usage as well as more positive attitudes towards such apps as in comparison to non-users. In terms of *Perceived usefulness*, fitness apps were seen as helpful in initiating physical activity, increasing fitness levels and promoting a healthy lifestyle. Specific training goals, self-discipline and motivation were mentioned as crucial conditions.

#### Barriers: Lacking ease of use & privacy concerns

Two central themes revealed as barriers towards fitness app usage: First, regarding the *Ease of use*, a lack of practical handling and simplicity was identified as Barrier to fitness app usage by Wichmann et al. [53]. More specifically, manual data entry and the permanent carrying of a mobile phone for the app’s purposes were perceived as hindrances. Second, *Privacy concerns* were addressed by two quantitative and both qualitative studies: In Mackert et al. [51], older people (not specified) showed significantly greater privacy concerns regarding fitness and nutrition app usage (ß = -.111, *P* < .001; ß = -.092, *P* < .001). In contrast, Bol et al. [54] did not find any significant relation between data protection concerns and fitness or nutrition app acceptance. In Cabrita et al. [55], one participant dropped out during the study, due to privacy concerns. More explicitly, Wichmann et al. [53] found *Privacy concerns* as barrier towards fitness app usage. Participants were concerned with potential data use by health insurance companies or for commercial purposes. However, if a particular fitness app is recommended, the use of such an app would come into question despite such data protection concerns. The focus groups also revealed a certain technologization skepticism. In this context, fitness apps were seen as disadvantageous, as their use would inhibit the ability to self-perceive bodily functions. In addition, a desire to determine the intensity of the activity autonomously rather than being "dictated" by a smartphone app was expressed.

#### Wishes and expectations

Prior to app usage, participants of the case study by Cabrita et al. [55] were asked about their *Wishes and expectations* regarding nutrition and fitness apps. Expectations towards nutrition apps were not shared, yet “most participants” [55] expected to adapt their physical activity to the feedback received when using a fitness app. Moreover, resulting from our thematic analysis, several wishes of fitness and nutrition apps were identified and could be clustered as *Monitoring*, *Motivating and Social exchange features* (see Table 4). Similarly, Wichmann et al. [53] found wishes towards fitness apps with respect to specific monitoring (e.g., automatic, non-manual activity tracking) and feedback functions. Such wishes were similar for both, fitness app users and non-users.

## Discussion

In order to support healthy aging, the promotion of beneficial lifestyles such as a healthy diet and sufficient exercise in middle and older age is vital. To meet the global challenges associated with the demographic shift, mHealth services may be effective and cost-efficient tools to support people transitioning to old(er) age in maintaining and/or achieving a healthy lifestyle, which may prevent or delay (multi-)morbidity and functional decline and thereby improve the quality of life [6, 13, 58, 59]. While research findings have shown beneficial effects of app-based interventions on dietary habits and physical activity [7, 9, 10, 13, 16, 60], a lack of consistent and conclusive evidence remains, indicating a need for further high quality (longitudinal) studies that demonstrate the efficacy of mHealth apps in promoting healthier habits among ageing populations [9, 61]. To adequately investigate the effectiveness of mHealth apps, it seems worthwhile to establish a comprehensive body of knowledge regarding middle-aged *and* older adults’ interest in and general attitudes towards such apps as well as their actual usage behavior. Therefore, this systematic mixed studies review dealt with the question in how far the specific, yet heterogeneous target group of middle-aged and older people accepts mobile nutrition and fitness apps and which influencing factors and social determinants shape this acceptance. Following an exploratory approach, we not only considered studies with various research designs for synthesis, but also the broad range of definitions and operationalizations of app acceptance [cf. 26] that inductively emerged during the screening process.

Overall, the existing body of literature regarding our review questions proved to be sparse. While there is a great number of studies investigating acceptance of mHealth technology in general or regarding specific health outcomes [6, 7, 62, 63] and general or younger population groups [8, 64, 65], seven (non-interventional) studies only could be identified that specifically deal with nutrition and fitness apps in middle-aged and/or older populations. Among the reviewed studies, just two studies focused on nutrition and/or fitness apps [52, 57]; the others dealt with health apps in general, including those specific ones. While the Swiss study [57] and the qualitative studies [53, 55] exclusively addressed middle-aged and older adults, the other ones involved these age groups in their research on general adult populations.

### Study quality

Applying the AXIS and MMAT quality appraisal criteria, the included studies showed divergent levels of quality. Three studies [52, 54, 56] met the (vast) majority, whereas one study [51] did not meet a substantial number of the AXIS quality criteria (see S4 Appendix). Most shortcomings were identified in the methods sections. Notably, none of the studies reported on and/or took measures to address non-responders. If no measures to deal with non-responders were taken, concerns regarding potential non-response bias may raise. The MMAT appraisal of the qualitative study by Cabrita et al. [55] revealed inadequacies concerning the data collection process and the analysis and interpretation of the data material. Little data material was provided to support the findings, which may render some interpretative statements questionable and hamper transparent, repeatable research. The paper by Wichmann et al. [53] showed a relatively high study quality according to the MMAT appraisal tool. With respect to both the quantitative studies and the qualitative ones, it remains unclear whether the identified shortcomings reflect methodological practice; the least, these stress a need for increased reporting quality.

### The digital divide and social determinants of nutrition and fitness app acceptance

Overall, the synthesized findings seem to point towards the existence of a *digital divide*. Consistent with research on general mHealth technology acceptance [17, 22, 63, 66], this systematic mixed studies review indicates that this generation gap partially manifests in lower usage frequencies, on the one hand, and in differently pronounced attitudes towards nutrition and/or fitness apps among middle-aged and older compared to younger age groups, on the other hand. Lower usage rates of middle-aged and older adults were shown in all quantitative studies. In the qualitative studies, none of the participants interviewed by Cabrita et al. [55] had used mHealth apps before, while Wichmann et al. [53] had specifically recruited nonusers as well as users of fitness apps. Contrasting participants’ usage rates across studies, a mixed picture emerged: The quantitative studies found considerable proportions of middle-aged and especially “older adults” (not always specified within studies; not consistently defined across studies), who (had previously) used nutrition and/or fitness apps–albeit to largely varying extents. Remarkably, the two studies with the oldest samples showed the lowest usage rates [57] resp. no prior usage [55]. This underscores age group differences that have been previously demonstrated with respect to general mHealth apps [21, 22]. Surprisingly, while the Austrian study by Naszay et al. [56] was based on the youngest sample (mean age, SD = 36.9 yrs., +/-1.2), a relatively low proportion of fitness and nutrition app usage was found. In contrast, the Dutch study by Bol et al. [54]–with a sample of smart device owners aged 50 years, on average,–showed by far the greatest usage rates. Here, several sources of bias may have played a role: For example, not only the relatively large age range (r = 18–89 yrs.) [54] but also further sample characteristics, such as the socio-cultural background, may have outplayed age group effects.

Regarding attitudes towards app usage, some of the synthesized results suggest middle-aged and older people to be less interested in such apps [55, 56], perceive them as less useful [51, 55], and to show greater privacy concerns [51]. In their qualitative study on older (non-)fitness app users, Wichmann et al. [53] found various barriers towards app usage, including privacy concerns and technologization skepticism as well as a lack of perceived ease of use. Such attitudes have previously been observed as barriers to the adoption and/or continued use of general technologies, whereby privacy concerns and trustworthiness are among the greatest concerns older people hold against technologies [18, 42, 66, 67]. Moreover, compared to nutrition apps, fitness apps appeared to find a somewhat greater acceptance among middle-aged and older users. Reasons for such preferences were not provided by the quantitative studies, whereas Cabrita et al. [55] revealed more pronounced reasons for the participants’ reluctance and rejection of nutrition app compared to fitness app usage, and specifically showed a lack in *Perceived usefulness*. Drawing on acceptance studies of general mHealth tools, these findings may also be related to the *Perceived ease of use* resp. the *Usability*, which is one of the major facilitators of mHealth app usage for general populations [42] as well as for older age groups [68]. Its associated factors such as easy and automatic handling and simple tracking appear decisive for the initiation and/or sustainability of health app usage [40, 42, 64]. Typical features of fitness apps such as step counts are perceived as easy to use, e.g. due to their full-automatic functioning. In contrast, food tracking, which is a crucial nutrition app feature, often requires detailed entries or similar actions (e.g. taking a photo). Thus, users (of all age groups) often perceive a limited usability because such apps resp. app features are too complex and time-consuming, among others [42]. However, it is crucial to mention that the *Perceived ease of use* is among the acceptance dimensions that have most frequently been studied [44] and its importance may also be related to the deductive application of particular acceptance definitions.

Our review findings highlight that there is little robust evidence on social determinants and contextual factors influencing nutrition and fitness app acceptance among middle-aged and older people. Previous evidence suggests socioeconomic-related, health-related, and literacy-related disparities in app adoption [66, 69–71]. Some of the included quantitative studies assessed few such covariates, yet did not stratify such findings for different age groups. Regarding gender, four studies showed differences, with men having shown to be more likely to use fitness apps [54, 57]. In contrast, findings by Bol et al. [54] as well as by König et al. [52] showed women to use nutrition apps more often as in comparison to men. Consistent results were found in relation to the influence of the socioeconomic background; however, this was only investigated by two studies [51, 54]. In both cases, participants with a higher social status and/or income were more likely to use fitness and nutrition apps. The qualitative studies [53, 55] did not provide further insights regarding factors shaping nutrition and/or fitness app acceptance, since characteristics such as age and gender were not reflected when citing interviewees. While these findings on the digital divide and social determinants regarding nutrition and fitness app acceptance are still tenuous, they may–if substantiated by further in-depth research–not only inform targeted app development, but also guide future health promotion strategies. Therefore, further research is needed that specifically and with methodological soundness deals with older population groups. In order to be able to draw more robust conclusions about the acceptance of nutrition and fitness apps among people transitioning to older age, future research activities should thereby explicitly define and thoroughly operationalize this rather broad age group and investigate age effects as well as their associations with other (social) determinants within this heterogeneous group of people. Further research needs that crystallized from our synthesis are discussed below.

### The need for longitudinal study designs

Notably, all the included quantitative studies were cross-sectional, examining acceptance as a snapshot. Rather than a static state, acceptance is a dynamic process influenced by various internal and external factors [72, 73]. Health app usage should therefore be addressed by examining potential changes in the acceptance of middle-aged and older people over time. It thus seems promising to separately and longitudinally analyse the (pre-)initiation, translation-of-intention-into-action, and maintenance phases of app usage [74, 75]. However, such research is still lacking, for example, with respect to mHealth applications: Based on their recent scoping review on scientific definitions and operationalizations of mHealth technology acceptance, Nadal et al. [26] stress the importance to account for the processual character of acceptance and to distinguish between different stages of technology acceptance. Differentiating the so far mostly synonymously used terminologies *Acceptability*, *Acceptance* and *Adoption*, the authors propose the Technology Acceptance Lifecycle (TAL). The TAL depicts a continuum between the Preadoption and Postadoption phase, which encompasses *Pre-use acceptability* on the one hand, and *Initial use acceptance* and *Sustained use acceptance*, on the other hand [26]. Regarding the so-called *intention-behavior gap*, such different stages may differ in so far, as initial interest may occur, but actual usage does not follow or adherence diminish over time [76]. Reasons for usage stops and hesitance to or rejection of app usage may be different in nature and influenced by distinct barriers and should hence be examined independently. Recent investigations on general populations (not age group-specific) have addressed reasons for disrupted and discontinued app usage [77, 78], thereby accounting for barriers to sustained usage acceptance. Still, most research on (mHealth) technology usage focuses on the pre-initiation phase, neglecting, for instance, investigations of sustained usage acceptance [73]. Resulting from this systematic review, this also holds true for research on middle-aged and older adults’ nutrition and fitness app acceptance. Two studies only [52, 53] (partially) accounted for a processual character of app acceptance: König et al. [52] addressed different fitness and nutrition app “adoption stages” [52] and could identify specific facilitators and barriers resp. “motivational stage differences” [52]. Despite the findings’ important insightful value, its explanatory power may be limited by the cross-sectional research design.

In Wichmann et al. [53], the comparative exploration of fitness app acceptance of non-users and users aged 50 years and older provides first qualitative insights regarding the facilitators and barriers associated with pre-adoption. Moreover, the Dutch qualitative study [55] observed positive changes in interviewees’ attitudes toward specific app features. Particularly regarding fitness apps, previous reluctance or rejection of app use changed over the course of the (case) study. This seems related to the fact that barriers to initial usage were overcome, since app use was externally induced and encouraged by the researchers. Both positive attitudes and initial adoption have a significant impact on usage adherence [30]. Therefore, overcoming this first barrier represents a crucial moment for subsequent behavior change. The authors [55] underline this with special regard to specific fitness and nutrition app features. Yet, due to their quasi-experimental research design, their results do not reflect real-life acceptance and associated mechanisms, and participation in the study itself may indicate a certain level of interest in mHealth technology. Thus, future quantitative as well as qualitative studies are needed that longitudinally examine different stages of fitness and nutrition app adoption.

### The need for comprehensive investigations of app acceptance

While none of the included studies explicitly applied a theoretical acceptance framework such as the TAM [30] or the UTAUT (2) [31, 79], a few of such dimensions were inductively retrieved by the thematic analysis. Most dimensions of app acceptance were examined by the qualitative studies [53, 55], which is certainly related to the study design.

In all quantitative studies, *Actual usage behavior*, mostly measured by the frequency of use, was investigated. *Attitudes* towards such apps also surfaced as an overarching theme from all included studies, but the variance is limited because only few associated factors were examined. The study by Mackert et al. [51] was the only one that assessed the two influencing factors *Perceived usefulness* and *Ease of use*, as proposed by the TAM, for instance. This is surprising, since, as mentioned above, these factors are–with respect to technology acceptance in general–the most frequently acceptance dimensions investigated [42, 44].

*Facilitating conditions* were addressed by analyses of the reasons for app usage [57] and the reluctance or rejection to do so [51, 53, 55]. Thereby, as the only study assessing psychological factors, Mackert et al. [51] investigated the influence of health literacy on app usage and showed a positive correlation. Addressing usage experiences, Cabrita et al. [55] found that non-usage and reluctance to use were related to the participants’ absence of knowledge on and familiarity with mHealth apps. Yet, during the first interview, interest in usage was seemingly elevated, particularly for fitness apps. The interview setting itself may hence have functioned as a facilitating condition. Cabrita et al. [55] further revealed their interviewees’ wishes and expectations that could facilitate future app adoption and/or continued use, with their findings being consistent with research on younger populations [71, 78]. The specific study design used by Cabrita et al. [55] and potentially arising effects such as social desirability may have had an impact on their findings. To validate those, future qualitative and quantitative studies are worthwhile that explore and measure reasons for reluctance or rejection of app usage in older populations.

*Behavioral intention*, which depicts a key factor shaping technology use according to both the TAM and the UTAUT 2, was only assessed by one study [55]. Moreover, applying the UTAUT 2 framework, various dimensions of app acceptance have hardly or not at all been addressed. For example, dimensions such as “social influence” as well as “hedonic motivations” and contextual influencing factors were rarely dealt with [79].

With respect to general mHealth tools and/or general population groups, there is a growing body of literature addressing barriers and facilitators of app acceptance in a more comprehensive and multidimensional manner, involving emotional, personal and social influencing factors may play a greater role than utilitarian ones [35, 42, 80]. Based on a systematic review, Perski et al. [80] have developed a holistic conceptual framework of diverse (in)direct factors influencing the use of digital behavior intervention tools–such as fitness and nutrition apps. Thereby, this framework accounts for contextual factors on the micro-, meso- and macro-level, involving psychological (e.g. motivation), demographic (e.g. age, education), social (e.g. norms, media influence) and physical ones (e.g. policy, healthcare system). So far, such an encompassing approach has neither been applied to nutrition and/or fitness acceptance, nor to particular population groups such as older adults.

The results of our systematic review support this, since contextual factors were–if at all–focused on the individuals’ experiences or demographic characteristics. Psychological aspects such as motivational processes [cf. 78] have hardly been examined and societal or cultural influences, amongst others, have not yet been considered with respect to our target group.

Hence, our synthesis emphasizes that–within the scarce body of literature–only fragments of nutrition and fitness app acceptance among middle-aged and older adults have so far been understood. To improve the heterogeneous target group’s acceptance towards nutrition and fitness apps, tailored measures rather than “one-size-fits all” ones are vital [42], calling for an in-depth understanding of the lifeworlds, specific needs and diverse contexts of middle aged and older people [23, 25, 80]. Hence, future research is necessary that not only applies validated theoretical frameworks but also empirically expands those [cf. 80] to illuminate the complex dimensions of acceptance. To do so, taking interdisciplinary perspectives is important, so that, for instance, psychological decision-making processes and socio-cultural contexts and influencing factors can integratively be measured and understood, so that a holistic explanative picture emerges. Moreover, comprehensive investigations are needed with a clear focus on adults transitioning into old(er) age, involving more profound analyses of potential influencing factors and their interrelations.

### Strengths and limitations

The results of this systematic review are of value for future research directions, based on which the development, implementation and promotion of prevention and intervention strategies to improve older people’s diets and physical activity may be informed. The mixed studies review approach allowed for a comprehensive search involving diverse study designs. The narrative synthesis and thematic analysis facilitated an inductive approach and integration of the study results, by means of which relevant findings could be extracted and synthesized without being guided by preceding assumptions. Since our search strategy was not based on an a priori definition of acceptance, a relatively broad and unbiased search for potentially relevant articles could be realized. Despite these efforts, the predominantly incoherent, indistinct use of terminologies, definitions, as well as operationalizations of mHealth acceptance [cf. 26], may have constrained the comprehensiveness of the search results.

Given the tenuous state of research and the studies’ different levels of quality, general inferences on the review questions should yet be drawn with caution. One factor limiting the external validity of the synthesized results concerned the heterogeneous study foci and target populations and thus the correspondingly great variance of the samples in terms of age ranges, on the one hand, and regarding app usage, on the other hand. Accordingly, the conclusiveness with regard to adults aged 50 years and older showed to be limited and cross-study comparability was restricted, implying a limitation of the explanatory power of the reviewed evidence.

The results of this review may not readily be applied to different socio-cultural populations, as the studies geographically focused on western industrialized regions, especially German-speaking countries. In addition, searching in three databases may have restricted the total search results. The same applies to the exclusion of non-original articles, grey literature as well as articles published in languages other than English, French, and German.

## Conclusions

As this systematic mixed studies review highlights, the current state of knowledge on the review question appears to be sparse and several research gaps exist, underscoring the need for future studies to examine older adults’ acceptance of nutrition and/or fitness apps in greater breadth as well as depth. While still inconclusive, the current literature yet points at the existence of a *digital divide*, i.e., of a relatively low acceptance of middle-aged and older adults towards nutrition and fitness apps compared to younger ones. Therefore–in line with research on general mHealth apps–future nutrition and fitness apps should target the distinct needs of the heterogeneous group of adults aged 50 years and older. This calls for the meaningful involvement of this target group, not only in terms of participatory research, but also building on this in the process of app development. In doing so, it is important to pick up people from their life worlds, i.e. to first determine their individual contextual conditions such as motivation levels and to then target them specifically. Finally, effective strategies need to be established that reduce barriers to initial usage as well as promote adherence among middle-aged and older adults, and thus facilitate an effective support to promote healthy aging. Thereby, it seems worthwhile to address people before the transition into old(er) age as to “bridge” the effect of the so-called digital divide.

## Supporting information

S1 Appendix PRISMA 2009 checklist. (PDF)

S2 Appendix Search strategies (EMBASE & medline). (PDF)

S3 Appendix (PDF)

S4 Appendix Quality appraisal (AXIS & MMAT). (PDF)

S5 Appendix (PDF)

## Abbreviations

AXIS: Appraisal Tool for Cross-Sectional Studies
mHealth: mobile health
MMAT: Mixed Methods Appraisal Tool
PRISMA: Transparent Reporting of Systematic Reviews and Meta-analyses
PROSPERO: International Prospective Register of Systematic Reviews
SD: Standard deviation
TAM: Technology Acceptance Model
UTAUT: Unified Theory of Acceptance and Use of Technology
