# Supplementary material for: Middle-aged and older adults’ acceptance of mobile nutrition and fitness apps: A systematic mixed studies review
Source: PLoS One. 2022 Dec 15;17(12):e0278879. doi: 10.1371/journal.pone.0278879 (PMC9754197; doi:10.1371/journal.pone.0278879)
Supplement: S2 Appendix — (PDF) [file pone.0278879.s002.pdf]

First search strategy for EMBASE

Database: Embase Classic+Embase <1947 to 2020 December 03>
Search Strategy:
----
1 Mobile Applications/ (11330)
2 ((Mobil* adj2 Application*) or (Mobil* adj2 App?) or (Portable adj2 Electronic App*) or (Portable adj2 Software App*)).ab,ti. (8593)
3 exp mobile phone/ (31096)
4 personal digital assistant/ (1533)
5 (handheld adj1 (computer? or pc?)).ab,ti. (532)
6 cell$ phone.ab,ti. (3279)
7 mobile phone?.ab,ti. (10670)
8 smartphone?.ab,ti. (16421)
9 palmtop computer?.ab,ti. (98)
10 (tablet adj3 (device? or comput$)).ab,ti. (2275)
11 1 or 2 or 3 or 4 or 5 or 6 or 7 or 8 or 9 or 10 (50725)
12 exp exercise/ (380385)
13 exp fitness/ (41280)
14 exp sport/ (180091)
15 exp Endurance/ (25295)
16 exercis*.ab,ti. (425441)
17 sport*.ab,ti. (106522)
18 ((physical or motion) adj5 (fitness or therapy or therapies)).ab,ti. (54935)
19 12 or 13 or 14 or 15 or 16 or 17 or 18 (774133)
20 geriatric nutrition/ (917)
21 exp diet/ (382655)
22 nutri*.ab,ti. (552083)
23 diet.ab,ti. (468271)
24 caloric intake/ (64963)
25 food.ab,ti. (557532)
26 eating.ab,ti. (105475)
27 20 or 21 or 22 or 23 or 24 or 25 or 26 (1526127)
28 19 or 27 (2219820)
29 11 and 28 (5729)
30 limit 29 to (embryo <first trimester> or infant <to one year> or child <unspecified age> or preschool child <1 to 6 years> or school child <7 to 12 years> or adolescent <13 to 17 years>) (974)
31 29 not 30 (4755)
32 limit 31 to animal/ (64)
33 31 not 32 (4691)
34 limit 33 to yr="2019-2020" (1656)
35 limit 34 to exclude medline journals (225)

****

Second search strategy for EMBASE

Database: Embase <1974 to 2019 January 07>
Search Strategy:
----
1 Mobile Applications/ (6058)
2 ((Mobil* adj2 Application*) or (Mobil* adj2 App?) or (Portable adj2 Electronic App*) or (Portable adj2 Software App*)).ab,ti. (4859)
3 exp mobile phone/ (21506)
4 personal digital assistant/ (1294)
5 (handheld adj1 (computer? or pc?)).ab,ti. (513)
6 cell$ phone.ab,ti. (2743)
7 mobile phone?.ab,ti. (8177)
8 smartphone?.ab,ti. (9499)
9 palmtop computer?.ab,ti. (97)
10 (tablet adj3 (device? or comput$)).ab,ti. (1719)
11 1 or 2 or 3 or 4 or 5 or 6 or 7 or 8 or 9 or 10 (34249)
12 exp exercise/ (302573)
13 exp fitness/ (35068)
14 exp sport/ (144583)
15 exp Endurance/ (21658)
16 exercis*.ab,ti. (350831)
17 sport*.ab,ti. (86205)
18 ((physical or motion) adj5 (fitness or therapy or therapies)).ab,ti. (43069)
19 12 or 13 or 14 or 15 or 16 or 17 or 18 (627488)
20 geriatric nutrition/ (809)
21 exp diet/ (287174)
22 nutri*.ab,ti. (445782)
23 diet.ab,ti. (376013)
24 caloric intake/ (55837)
25 food.ab,ti. (450501)
26 eating.ab,ti. (87442)
27 20 or 21 or 22 or 23 or 24 or 25 or 26 (1226045)
28 19 or 27 (1786426)
29 11 and 28 (3548)
30 limit 29 to (embryo <first trimester> or infant <to one year> or child <unspecified age> or preschool child <1 to 6 years> or school child <7 to 12 years> or adolescent <13 to 17 years>) (600)
31 29 not 30 (2948)
32 limit 31 to animal/ (47)
33 31 not 32 (2901)
34 limit 33 to yr="2008 - 2019" (2819)
35 limit 34 to exclude medline journals (363)
****

First search strategy for Medline

Database: Ovid MEDLINE(R) ALL <1946 to December 03, 2020>
Search Strategy:
----
1 Mobile Applications/ (6611)
2 ((Mobil* adj2 Application*) or (Mobil* adj2 App?) or (Portable adj2 Electronic App*) or (Portable adj2 Software App*)).ab,ti. (6643)
3 exp Cellular Phone/ (11095)
4 Computers, Handheld/ (3677)
5 (handheld adj1 (computer? or pc?)).ab,ti. (440)
6 cell$ phone.ab,ti. (2333)
7 mobile phone?.ab,ti. (8536)
8 smartphone?.ab,ti. (12259)
9 palmtop computer?.ab,ti. (92)
10 (tablet adj3 (device? or comput$)).ab,ti. (1448)
11 1 or 2 or 3 or 4 or 5 or 6 or 7 or 8 or 9 or 10 (37622)
12 exp exercise/ (200829)
13 exp Physical Fitness/ (30945)
14 exp sports/ (186396)
15 exp Physical Endurance/ (34015)
16 exercis*.ab,ti. (299970)
17 sport*.ab,ti. (77248)
18 ((physical or motion) adj5 (fitness or therapy or therapies)).ab,ti. (35606)
19 12 or 13 or 14 or 15 or 16 or 17 or 18 (546380)
20 Elder Nutritional Physiological Phenomena/ (229)
21 exp diet/ (285142)
22 nutri*.ab,ti. (411823)
23 diet.ab,ti. (329247)
24 energy intake/ (41148)
25 food.ab,ti. (431788)
26 eating.ab,ti. (76623)
27 20 or 21 or 22 or 23 or 24 or 25 or 26 (1167258)
28 19 or 27 (1656502)
29 11 and 28 (4235)
30 limit 29 to ("all infant (birth to 23 months)" or "all child (0 to 18 years)" or "newborn infant (birth to 1 month)" or "infant (1 to 23 months)" or "preschool child (2 to 5 years)" or "child (6 to 12 years)" or "adolescent (13 to 18 years)" or "young adult (19 to 24 years)" or "adult (19 to 44 years)" or "young adult and adult (19-24 and 19-44)") (1765)
31 29 not 30 (2470)
32 limit 31 to animals/ (56)
33 31 not 32 (2414)
34 limit 33 to yr="2019 - 2020" (965)

****

Second search strategy for Medline

Datenbank: Ovid MEDLINE(R) and Epub Ahead of Print, In-Process & Other Non-Indexed Citations and Daily <1946 to January 07, 2019>
Suchstrategie:
----
1 Mobile Applications/ (3635)
2 ((Mobil* adj2 Application*) or (Mobil* adj2 App?) or (Portable adj2 Electronic App*) or (Portable adj2 Software App*)).ab,ti. (3914)
3 exp Cellular Phone/ (9113)
4 Computers, Handheld/ (3294)
5 (handheld adj1 (computer? or pc?)).ab,ti. (433)
6 cell$ phone.ab,ti. (2027)
7 mobile phone?.ab,ti. (6650)
8 smartphone?.ab,ti. (7170)
9 palmtop computer?.ab,ti. (91)
10 (tablet adj3 (device? or comput$)).ab,ti. (1086)
11 1 or 2 or 3 or 4 or 5 or 6 or 7 or 8 or 9 or 10 (26221)
12 exp exercise/ (173218)
13 exp Physical Fitness/ (26607)
14 exp sports/ (168135)
15 exp Physical Endurance/ (31262)
16 exercis*.ab,ti. (267098)
17 sport*.ab,ti. (65505)
18 ((physical or motion) adj5 (fitness or therapy or therapies)).ab,ti. (30724)
19 12 or 13 or 14 or 15 or 16 or 17 or 18 (480656)
20 Elder Nutritional Physiological Phenomena/ (211)
21 exp diet/ (257436)
22 nutri*.ab,ti. (355446)
23 diet.ab,ti. (290723)
24 energy intake/ (38342)
25 food.ab,ti. (367017)
26 eating.ab,ti. (65872)
27 20 or 21 or 22 or 23 or 24 or 25 or 26 (1019936)
28 19 or 27 (1451507)
29 11 and 28 (2678)
30 limit 29 to ("all infant (birth to 23 months)" or "all child (0 to 18 years)" or "newborn infant (birth to 1 month)" or "infant (1 to 23 months)" or "preschool child (2 to 5 years)" or "child (6 to 12 years)" or "adolescent (13 to 18 years)" or "young adult (19 to 24 years)" or "adult (19 to 44 years)" or "young adult and adult (19-24 and 19-44)") (1091)
31 29 not 30 (1587)
32 limit 31 to animals/ (39)
33 31 not 32 (1548)
34 limit 33 to yr="2008 - 2019" (1505)
****
