# Supplementary material for: Middle-aged and older adults’ acceptance of mobile nutrition and fitness apps: A systematic mixed studies review
Source: PLoS One. 2022 Dec 15;17(12):e0278879. doi: 10.1371/journal.pone.0278879 (PMC9754197; doi:10.1371/journal.pone.0278879)
Supplement: S3 Appendix — (PDF) [file pone.0278879.s003.pdf]

A	B	C	D	E	F	G	H
**AUTHORS**	**GENERAL INFORMATION**	**Title**	**Journal**	**Year of publication**	**Geographical context**	**RESEARCH FOCI RELEVANT TO THE REVIEW QUESTION**	**Research objectives/ Aims/ Questions**
**Bol, Helberger & Weert**		Differences in mobile health app use: A source of new digital inequalities?	The Information Society	2018	Amsterdam, The Netherlands		major research aims:<br>to examine possible differences in mobile phone usage based on sociodemographic factors, ehealth literacy and privacy concerns<br>to examine if these differences differ depending on the type of app used
**Cabrita, Tabak, Vollenbroek-Hutten**		Older Adults' Attitudes Toward Ambulatory Technology to Support Monitoring and Coaching of Healthy Behaviors: Qualitative Study	JMIR Aging	2019	Enschede, The Netherlands		aim: to investigate current practices in health management as well as (changes in) attitudes of older independently living adults regarding mHealth technologies (focus on the domains: nutrition, PA, well-being, cognitive functioning) for health management (including wishes and expectations) - following a four week actual usage phase within the scope of a case study
**König, Sproesser, Schupp & Renner**		Describing the Process of Adopting Nutrition and Fitness Apps: Behavior Stage Model Approach	JMIR Mhealth and UHealth	2018	Konstanz, Germany		major research aims:<br>- examine different behavioral stages of adopting nutrition and fitness apps<br>- look at sociodemographic, behavioural & psychological characteristics of people in different stages<br>--> explore transition barriers between the different stages
**Mackert, Mabry-Flinn, Champlin, Donovan & Pounders**		Health Literacy and Health Information Technology Adoption: The Potential for a New Digital Divide	Journal of Medical Internet Research	2016	Austin, United States		major research aim:<br>examine relation between health literacy and use of health information technology,<br>4 research questions:<br>- relation between health literacy and HIT usage<br>- association between health literacy and ease of use and perceived usefulness of HIT<br>- association between health literacy and perceived privacy of HIT<br>- relation between health literacy and perceived trust in health related institutions
**Seifert, Schlomann, Rietz & Schelling**		The use of mobile devices for physical activity tracking in older adults' everyday life	Digital Health	2017	Zurich, Switzerland		major research aim:<br>to examine the usage (and reason for usage) of mobile physical activity trackers amongst older people (50+ years)<br>--> 3 research questions:<br>- number of older physical activity tracker users<br>- users compared to non-users<br>- reasons for physical activity tracking device usage
**Naszay, Stockinger, Jungwirth & Haluza**		Digital age and the Public eHealth Perspective: Prevailing health app use among Austrian Internet users	Informatics for Health & Social Care	2018	Vienna, Austria		major research aim:<br>to examine differences in digital natives' and digital immigrants' views on eHealth (including health apps) and telemedicine

A	I	J	K	L
**AUTHORS**	**Hypotheses/ Rationales**	**STUDY DESIGN & METHODS RELEVANT TO THE REVIEW QUESTION**	**Sampling**	**Methodology & Data gathering methods**
**Bol, Helberger & Weert**	mobile health apps might increase the inequality in the health care system instead of decreasing it, therefore examining if factors related to the use of Internet technology apply to the use of mobile health apps as well; different types of mobile health apps might have different user groups in terms of sociodemographic factors		recruitment via the CentERdata's LISSPANEL --> a representative sample of the Dutch population	**cross-sectional study:**<br>Standardized questionnaire via online survey<br>**measures:**<br>health app usage --><br>e-health literacy<br>privacy concerns<br>sociodemographic measures --> age, gender, educational level
**Cabrita, Tabak, Vollenbroek-Hutten**	encouraging older people's health self-management by means of ambulatory technology targeting health behaviors (e.g. nutrition / PA app) may support the prevention of functional decline.Therefore, the target group's needs should be better understood and addressed to increase user adherence.		older adults were recruited via:<br>1. local information markets to promote healthy behaviors in the region of Overijssel (NL) and<br>2. information sessions given to participants in the European Project - Personalized ICT Supported Service for Independent Living and Healthy Ageing (PERSSILLA)	**Qualitative study:**<br>**semi-structured interviews (pre and post* actual use of technology),** interviews took place at Roessingh research and Development (approx. 1 hour) and were conducted by two researchers following written informed consent; interviews were tape recorded and transcribed verbatim; interview guideline was divided into 5 sections: 1. general health management, 2. nutrition, 3. cognition, 4. physical function, 5. well-being;<br>Main interview questions posed:<br>1. What are you currently doing to manage your physical function, cognitive function, nutrition, and wellbeing?<br>2. What is your attitude towards monitoring physical function, cognitive function, nutrition, and wellbeing?<br>3. What are your wishes and expectations from technology to monitor physical function, cognitive function, nutrition, and well-being?<br>**case study - actual use of technology:**<br>participants received smartphones after the interview, a Fitbit Zip step counter (measuring steps and distance walked per day, sending feedback automatically; daily step goal was set 7500 steps),<br>a smart scale Withings 30 (automatic weight monitoring , changes in weight and BMI) &<br>the Activity Coach app (connected to the step counter and the weight scale);<br>participants were asked to use the technology at their own pace for a period of 4 weeks;<br>subsequently, a second semi-structured interview was conducted*
**König, Sproesser, Schupp & Renner**	hypothesis:<br>intuitive decision making style as a possible transistion barrier in non users		- data collection via the Konstanz Life Study--> ongoing longitudinal cohort study as part of the SMARTACT research projects<br>- participants from fourth wave of measurement in spring 2016 included<br>- particpants recruted through flyers, posters & newspaper articles + invitation via phone or email to participants of previous points of measurement	**cross-sectional study:**<br>standardized questionnaire via paper and pencil survey<br>**measures:**<br>--> sociodemographic characteristics<br>--> mobile device usage, nutrition and fitness app usage, frequency of usage<br>--> stage model for adoption process of health apps, based on PAMP, 5 different stages:<br>1 unengaged: never used a health app<br>2 decided to act: thought about it, but never used an app<br>3 decided not to act: thought about it,but don't need an app<br>4 acting: currently using an app and want to continue using it<br>5 disengaged: have used it, but don't anymore<br>--> preference for intution and deliberation in eating decision making<br>--> healthy eating style<br>--> BMI
**Mackert, Mabry-Flinn, Champlin, Donovan & Pounders**	HIT as an important tool to improve public health --> relation of health literacy to HIT use must be examined in order to develop HIT tools that are easy to use for everyone		participants recruited from an existing invitation-only research panel, participants received an email with a link to an online survey, were compensated for completing the survey	**Cross sectional study:**<br>Standardized questionnaire via online questionnaire<br><br>**measures:**<br>--> sociodemographics<br>--> health literacy via NVS Score<br>--> HIT use (fitness app, nutrition app, physical activity trackers, patient portals)<br>--> HIT perceptions (perceived ease of use & usefulness)<br>--> HIT privacy<br>--> trust in 4 institutions (government, media, technology companies, health care system)
**Seifert, Schlomann, Rietz & Schelling**	hypothesis:<br>- users are younger, more educated, male & more interested in new technology<br>- most common reasons for usage of physical acitivity tracker: self-monitoring, motivation to be more active		a random sample of Swiss residents who were 50 years or older was selsected from commercial AZ-Direct database (based on public phonebook)	**Cross sectional study:**<br>Standardized questionnaire via computer assisted telephone interview<br><br>**measures:**<br>--> usage of physical activity tracking devices (physical activity trackers, smartwatches & smartphone/tablet applications), divided into users (+mobile device usage), non users (but mobile device usage) & non users (without mobile device usage)<br>--> sociodemographic measures<br>--> reasons for physical activity tracking<br>--> interest in new mobile technology<br>--> exercise frequency<br>--> subjective physical health status
**Naszay, Stockinger, Jungwirth & Haluza**	hypothesis:<br>previous research suggests that digital natives are more likely to use ehealth --> digital gap betwieen digital natives & digital immigrants		recruitment via 3 different methods:<br>1. emails with a link through mailing lists of health-related professional associations<br>2. ad for the survey on facebook<br>3. invitations on health forums<br>--> also use of snowball recruitment: participants were asked to forward an invitation link to friends and family	**cross-sectional study:**<br>Standardized questionnaire via online survey<br>**measures:**<br>--> sociodemographic factors (--> division of participants in two subgroups: digital natives (aged 35 years or younger)<br>& digital immigrants (aged >35 years))<br>--> medical conditions<br>--> reasonability of electronic health information exchange between doctors and patients<br>--> information status on eHealth and telemedicine<br>--> usage of health apps<br>--> usefulness of collecting health data or behavior on apps<br>--> interest in health apps

A	M	N	O	P	Q	R	S
**AUTHORS**	**Analysis methods**	**SAMPLE AND PARTICIPANT CHARACTERISTICS**	**Sample size**	**Age**	**Sex**	**Participants' mobile phone ownership / app usage**	**Socio-economic status (SES)/ Income level**
**Bol, Helberger & Weert**	**statistical analysis:**<br>logistic regression analysis for relation between cumulative health app usage in and sociodemographic measures, e-health literacy and privacy concerns<br>logistic regression analysis for relation between categories of specific health app usage iand sociodemographic measures, e-health literacy and privacy concerns		N=1079	mean age = 50.32 +/- 16.35	n_f = 584<br>n_m = 495	only mobile phone owners were included	n.a.
**Cabrita, Tabak, Vollenbroek-Hutten**	**analsysis conducted by two researchers in two steps:**<br>**1. "concept-driven approach"** (p. 4)<br>-> deductive coding based on the categories: current practice, attitudes toward health management, wishes from technology<br>**2.** inductive coding of subthemes by means of **qualitative content analysis** (iterativ coding);<br>Atlas.ti 7.0		N=12;<br>second interview after case study: N=11	mean age = 69, r = 65-78	n_f = 7<br>n_m = 5	n_8 = possessed a smartphone, n=5 of which considered themselves as "advanced users"; None of the participants had prior experiences with PA apps or smart scale devices	n.a.
**König, Sproesser, Schupp & Renner**	**statistical analysis:**<br>- difference between fitness & nutrition app usage tested within subgroup of mobile phone owners<br>--> one-way analyses of variance, post hoc analyses with Bonferri corrections, Levene tests for precondition of homogenity of variances<br>- Welch tests and Games-Howell post hoc test for differences in age & BMI for nutrition app adoption stages and age differences between fitness app adoption stages<br>- chi square tests for gender differences<br>- adopting stage differences for intuition & liberation analysed with mixed ANOVAs		N=1215	mean age= 41.11 +/- 17.56	n_f = 783<br>n_m = 432	mobile phone owners and non-owners were included	n.a.
**Mackert, Mabry-Flinn, Champlin, Donovan & Pounders**	**statistical analysis:**<br>descriptive statistics of sociodemographic measures<br>health literacy x HIT cross tabulation<br>hierarchial linear regression analysis of each type of HIT use & privacy in relation to NVS Score & sociodemographics		N=4974	mean age = 43.5 +/- 16.7	n_f = 2872<br>n_m = 2102	mobile phone owners and non-owners were included	<$10000 n = 230<br>$10000-$49999 n = 1908<br>$50000-$99999 n = 1764<br>>$100000 n = 1068
**Seifert, Schlomann, Rietz & Schelling**	**statistical analysis:**<br>descriptive frequency distributions and group differences referring to age, gender and interest in technology using Cramér's V to answer the first two research questions, two logistic regressions based on three user groups to find interdependent factors for mobile device usage for physical activity tracking analysis of reasons for physical activity tracking in group users to answer third research question		N=1013	n_50-64 = 522<br>n_65-79 = 358<br>n ≥ 80 = 133	n_f = 538<br>n_m = 475	mobile phone owners and non-owners were included	n.a.
**Naszay, Stockinger, Jungwirth & Haluza**	**statistical analysis:**<br>descriptive statitics for qualitative values in means, SD & percentages<br>chi tests for differences between the age groups<br>binary logistics regression for association between interest in health apps and various factors		N=562	mean age = 36.9 +/- 15.2<br><br>n_digital natives = 305<br>n_digital immigrants = 257	n_f = 331<br>n_m = 231	mobile phone owners and non-owners were included	n.a.

A	T	U
**AUTHORS**	**Educational background**	**STUDY OUTCOMES RELEVANT TO THE REVIEW QUESTION**
**Bol, Helberger & Weert**	n_low =274<br>n_middle = 393<br>n_high = 410	**fitness app usage:**<br>older participants were significantly less likely to use fitness apps (b=0.03, SE=.01, p<.001; OR = 0.97; 95% CI 0.96-0.99)<br>**nutrition app usage:**<br>women more likely to use nutrition apps (b=-1.26, SE=.29, p<.001; OR=0.28; 95% CI 0.16-0.50) (only reported for overall sample)
**Cabrita, Tabak, Vollenbroek-Hutten**	Elementary schoon: n = 1<br>High school: n = 3<br>Vocational school: n = 6<br>University: n = 1<br>Missing: n = 1	**current practices in health management** (regarding physical activity (PA) and diet, not specific to apps) (N=12):<br>**PA:** n=11 mentioned PA health management<br>--> domain of health management assessed that was the one interviewees were most enganged with;<br>n=7 using bike for daily commuting; n=7 practiced sports at least 2 times per week, n=5 doing household to stay active;<br>**nutrition**: n=6 mentioned paying attention to eating habits on a daily basis (e.g. avoidance of sweets);<br>n=11 stated adoption of healthy dietary practices (e.g. cooking with low salt/sugar/carbs, taking small portion sizes, increase in vegetable consumption;<br>n=1 participant: not paying attention to daily eating, only cooks warm meals in company, otherwise all meals consist of bread<br>--> this interviewee was the only one who was overweight;<br>Reasons / motivations for (general) health management:<br>n=9: due to suffering from chronical condition (e.g. diabetes) that urge them to do so<br>n=4: wish for staying independent regarding daily acitvities<br>n=1: information abundance;<br>none referred to avoidance of disease; participants overall had a functional perspective on health<br>**Fitness/PA app usage:**<br>**Attitudes** (pre app usage in the context of the case study) (N=12):<br>--> at first, most interviewees were not open to monitoring their PA with technology;<br>after "explanation" (unclear what kind of explanation is referred to),<br>n=6 participants stated, they would maybe use an app or website to monitor thei PA;<br>n=5 still did not find important to monitor their PA using technology, since they rather rely on their feelings to decide whether the were active enough or not ("Look, when I'm fit and active, I feel good. Well, and I can feel that myself, I don't need to see that on one the computers, "you did this and that..." [72 yrs. old female] (Cabrita et al. 2019, p. 7));<br>n=1 participant thought monitoring PA by means of technology would make less attentive to the own body signals.<br>Wishes and expectations from technology (PA) (N=12):<br>--> participants expressed stronger wishes related to monitoring PA with technology than in any other health domain assessed (e.g. wellbeing, nutrition); CONTINUED*
**König, Sproesser, Schupp & Renner**	n.a.	**nutrition app usage:**<br>significant age differences between the different stages of behavioral adoption (F4252.00=16.85, p<.001, w2=.06) --> participants in stage 1 (uneganged) significantly older than partipants in stages 2 (decided to act), 4 (acting) & 5 (disengaged)<br>women more likely to be unengaged or disengaged (only reported for overall sample)<br>participants in stage 1 have a higher educational background than participants in stages 2 and 4 (only reported for overall sample)<br>**fitness app usage:**<br>significant age differences between the different stages of behavioral adoption (F4252.00=22.38, p<.001, w2=.08) --> participants in stage 1 (unengaged) significantly older than participants in all other 4 stages
**Mackert, Mabry-Flinn, Champlin, Donovan & Pounders**	n.a.	**nutrition app usage:**<br>older participants find nutrition apps significantly less easy to use (ß=-.145, p<.001) and sigificantly less useful (ß=-0.54, p<.001); significant association between educational background and ease of use for overall sample (ß=0.94, p<.001);<br>significant relation between different severity of health literacy and nutrition app usage for the overall sample (X21, N=4974=18.885, p<.001)<br>**fitness app usage:**<br>older participants find fitness apps significantly less easy to use (ß=-.204, p<.001) and sigificantly less useful (ß=-.106, p<.001);<br>significant association between educational background and ease of use for overall sample (ß=.017, p=<.001); significant relation between different severity of health literacy and fitness app usage for the overall sample (X21, N=4974=5.663, p=.02)<br>**privacy concerns:**<br>older participants are significantly more concerned about their privacy in relation to fitness (ß=-.111, p<.001) and nutrition app ß=-.092, p<.001) usage
**Seifert, Schlomann, Rietz & Schelling**	Obligatory school: n = 192<br>Secondary school: n = 569<br>Tertiary education: n = 245	**fitness app usage:**<br>15.1 % of all participants used at least one fitness app on their smartphone/tablet (24.1% of smartphone users, 26.5% of tablet users), half of them daily; group of the 50 - 64 years old participants significantly more often use fitness apps than those participants aged 56-79 years and those older than 80 years (V=0.10, p=.041);<br>men (V=.10, p=.006) & participants with interest in technology (V=.16, p<.001) significantly more likely to use fitness apps<br>**usage of health apps among smartphone and/or tablet owners:**<br>22% physical activity tracking, 16.5% documenting general well-being, 12.9% nutrition app, 3.9% control medication intake<br>**reasons for usage of physical activity tracking devices:**<br>65.8% "to track daily physical activity", 58.9% "to motivate myself to remain healthy", 21.5% "to exchange data on physical activity and health with friends", 17.2% "to document my data on physical activity and health for my physician", 13.7% "to track my sleep quality" --> younger users significantly more likely to track physical activity daily (V=.20, p=.017), older users significantly more likely to document data for physician (V=.30, p<.001)
**Naszay, Stockinger, Jungwirth & Haluza**	primary education n = 90<br>secondary education n = 191<br>tertiary education n = 281	**fitness app usage:**<br>--> significantly less health fitness app usage in the digital immigrant subgroup (95% CI=16.4-22.8, p=.005)<br>--> digital immigrants were significantly more likely to view the desirability of using exercise and nutrition apps in the future as low (p<.001)<br>**nutrition app usage:**<br>second most often used health apps by digital immigrants
