# Supplementary material for: Middle-aged and older adults’ acceptance of mobile nutrition and fitness apps: A systematic mixed studies review
Source: PLoS One. 2022 Dec 15;17(12):e0278879. doi: 10.1371/journal.pone.0278879 (PMC9754197; doi:10.1371/journal.pone.0278879)
Supplement: S4 Appendix — (PDF) [file pone.0278879.s004.pdf]

AXIS quality appraisal

Bol et al. (2017)					
	**QUESTION**	**YES**	**NO**	**Don't know**	**Not applicable**
Introduction					
1	Were the aims/objectives of the study clear?	x			
Methods					
2	Was the study design appropriate for the stated aim(s)?	x			
3	Was the sample size justified?	x			
4	Was the target/reference population clearly defined? (Is it clear who the research was about?)	x			

5	Was the sample frame taken from an appropriate population base so that it closely represented the target/reference population under investigation?	x			
6	Was the selection process likely to select subjects/participants that were representative of the target/reference population under investigation?			x	
7	Were measures undertaken to address and categorise non-responders?			x	
8	Were the risk factor and outcome variables measured appropriate to the aims of the study?	x			
9	Were the risk factor and outcome variables measured correctly using instruments/measurements that had been trialled, piloted or published previously?	x			
10	Is it clear what was used to determine statistical significance and/or precision estimates? (e.g. p-values, confidence intervals)	x			
11	Were the methods (including statistical methods) sufficiently described to enable them to be repeated?	x			
Results					

12	Were the basic data adequately described?	x			
13	Does the response rate raise concerns about non-response bias?		x		
14	If appropriate, was information about non-responders described?		x		
15	Were the results internally consistent?	x			
16	Were the results presented for all the analyses described in the methods?	x			
Discussion					
17	Were the authors' discussions and conclusions justified by the results?	x			
18	Were the limitations of the study discussed?	x			
Other					

19	Were there any funding sources or conflicts of interest that may affect the authors' interpretation of the results?		x		
20	Was ethical approval or consent of participants attained?	x			

**König et al. (2017)**					
	**QUESTION**	**YES**	**NO**	**Don't know**	**Not applicable**
Introduction					
1	Were the aims/objectives of the study clear?	x			
Methods					
2	Was the study design appropriate for the stated aim(s)?	x			
3	Was the sample size justified?	x			

4	Was the target/reference population clearly defined? (Is it clear who the research was about?)	x			
5	Was the sample frame taken from an appropriate population base so that it closely represented the target/reference population under investigation?	x			
6	Was the selection process likely to select subjects/participants that were representative of the target/reference population under investigation?	x			
7	Were measures undertaken to address and categorise non-responders?			x	
8	Were the risk factor and outcome variables measured appropriate to the aims of the study?	x			
9	Were the risk factor and outcome variables measured correctly using instruments/measurements that had been trialled, piloted or published previously?	x			
10	Is it clear what was used to determined statistical significance and/or precision estimates? (e.g. p-values, confidence intervals)	x			
11	Were the methods (including statistical methods) sufficiently described to enable them to be repeated?	x			

Results					
12	Were the basic data adequately described?	x			
13	Does the response rate raise concerns about non-response bias?	x			
14	If appropriate, was information about non-responders described?		x		
15	Were the results internally consistent?	x			
16	Were the results presented for all the analyses described in the methods?	x			
Discussion					
17	Were the authors' discussions and conclusions justified by the results?	x			
18	Were the limitations of the study discussed?	x			

Other					
19	Were there any funding sources or conflicts of interest that may affect the authors' interpretation of the results?		x		
20	Was ethical approval or consent of participants attained?	x			

**Naszay et al. (2017)**					
	**QUESTION**	**YES**	**NO**	**Don't know**	**Not applicable**
Introduction					
1	Were the aims/objectives of the study clear?	x			
Methods					
2	Was the study design appropriate for the stated aim(s)?	x			

3	Was the sample size justified?	x			
4	Was the target/reference population clearly defined? (Is it clear who the research was about?)	x			
5	Was the sample frame taken from an appropriate population base so that it closely represented the target/reference population under investigation?	x			
6	Was the selection process likely to select subjects/participants that were representative of the target/reference population under investigation?	x			
7	Were measures undertaken to address and categorise non-responders?			x	
8	Were the risk factor and outcome variables measured appropriate to the aims of the study?	x			
9	Were the risk factor and outcome variables measured correctly using instruments/measurements that had been trialled, piloted or published previously?	x			
10	Is it clear what was used to determined statistical significance and/or precision estimates? (e.g. p-values, confidence intervals)	x			
11	Were the methods (including statistical methods) sufficiently described to enable them to be repeated?	x			

Results					
12	Were the basic data adequately described?	x			
13	Does the response rate raise concerns about non-response bias?		x		
14	If appropriate, was information about non-responders described?		x		
15	Were the results internally consistent?	x			
16	Were the results presented for all the analyses described in the methods?	x			
Discussion					
17	Were the authors' discussions and conclusions justified by the results?	x			
18	Were the limitations of the study discussed?	x			

Other					
19	Were there any funding sources or conflicts of interest that may affect the authors' interpretation of the results?		x		
20	Was ethical approval or consent of participants attained?	x			

**Seifert et al. (2017)**					
	**QUESTION**	**YES**	**NO**	**Don't know**	**Not applicable**
Introduction					
1	Were the aims/objectives of the study clear?	x			
Methods					

2	Was the study design appropriate for the stated aim(s)?	x			
3	Was the sample size justified?		x		
4	Was the target/reference population clearly defined? (Is it clear who the research was about?)	x			
5	Was the sample frame taken from an appropriate population base so that it closely represented the target/reference population under investigation?	x			
6	Was the selection process likely to select subjects/participants that were representative of the target/reference population under investigation?	x			
7	Were measures undertaken to address and categorise non-responders?			x	
8	Were the risk factor and outcome variables measured appropriate to the aims of the study?	x			
9	Were the risk factor and outcome variables measured correctly using instruments/measurements that had been trialled, piloted or published previously?			x	
10	Is it clear what was used to determined statistical significance and/or precision estimates? (e.g. p-values, confidence intervals)	x			

11	Were the methods (including statistical methods) sufficiently described to enable them to be repeated?	x			
Results					
12	Were the basic data adequately described?	x			
13	Does the response rate raise concerns about non-response bias?	x	x		
14	If appropriate, was information about non-responders described?		x		
15	Were the results internally consistent?	x			
16	Were the results presented for all the analyses described in the methods?	x			
Discussion					
17	Were the authors' discussions and conclusions justified by the results?	x			

18	Were the limitations of the study discussed?	x			
Other					
19	Were there any funding sources or conflicts of interest that may affect the authors' interpretation of the results?		x		
20	Was ethical approval or consent of participants attained?	x			

Mackert et al. (2016)

	QUESTION	YES	NO	Don't know	Not applicable
Introduction					
1	Were the aims/objectives of the study clear?	x			
Methods					

2	Was the study design appropriate for the stated aim(s)?	x			
3	Was the sample size justified?		x		
4	Was the target/reference population clearly defined? (Is it clear who the research was about?)	x			
5	Was the sample frame taken from an appropriate population base so that it closely represented the target/reference population under investigation?			x	
6	Was the selection process likely to select subjects/participants that were representative of the target/reference population under investigation?			x	
7	Were measures undertaken to address and categorise non-responders?			x	
8	Were the risk factor and outcome variables measured appropriate to the aims of the study?	x			
9	Were the risk factor and outcome variables measured correctly using instruments/measurements that had been trialled, piloted or published previously?	x			
10	Is it clear what was used to determine statistical significance and/or precision estimates? (e.g. p-values, confidence intervals)	x			

11	Were the methods (including statistical methods) sufficiently described to enable them to be repeated?		x		
Results					
12	Were the basic data adequately described?	x			
13	Does the response rate raise concerns about non-response bias?			x	
14	If appropriate, was information about non-responders described?		x		
15	Were the results internally consistent?	x			
16	Were the results presented for all the analyses described in the methods?	x			
Discussion					
17	Were the authors' discussions and conclusions justified by the results?	x			

18	Were the limitations of the study discussed?	x			
Other					
19	Were there any funding sources or conflicts of interest that may affect the authors' interpretation of the results?		x		
20	Was ethical approval or consent of participants attained?			x	

MMAT quality appraisal

1. QUALITATIVE STUDIES	0.1. - Are there clear qualitative research questions?	0.2. - Do the collected data allow address the research question?
Cabrita et al. (2019)	YES	YES
	NO	NO
	CAN'T TELL / Somewhat	CAN'T TELL / Somewhat
	Screening question	*Screening question*
2. QUANTITATIVE STUDIES	0.1. - Are there clear quantitative research questions?	0.2. - Do the collected data allow address the research question?
Bol et al. (2018)	YES	YES
	NO	NO
	CAN'T TELL	CAN'T TELL
König et al. (2018)	YES	YES
	NO	NO
	CAN'T TELL	CAN'T TELL
Naszay et al. (2018)	YES	YES
	NO	NO
	CAN'T TELL	CAN'T TELL

Seifert et al. (2017)	YES	YES
	NO	NO
	CAN'T TELL	CAN'T TELL
Mackert et al. (2016)	YES	YES
	NO	NO
	CAN'T TELL	CAN'T TELL

1. QUALITATIVE STUDIES	1.1. - Is the qualitative approach appropriate to answer the research question?	1.2. - Are the qualitative data collection methods adequate to address the research question?	1.3. - Are the findings adequately derived from the data?
Cabrita et al. (2019)	YES	YES	YES
	NO	NO	NO
	CAN'T TELL / Somewhat	CAN'T TELL / Somewhat	CAN'T TELL / Somewhat
**2. QUANTITATIVE STUDIES**	**2.1. - Is the sampling strategy relevant to address the research question?**	**2.2. - Is the sample representative of the target population?**	**2.3. - Are the measurements appropriate?**
Bol et al. (2018)	YES	YES	YES
	NO	NO	NO
	CAN'T TELL	CAN'T TELL	CAN'T TELL
König et al. (2018)	YES	YES	YES
	NO	NO	NO
	CAN'T TELL	CAN'T TELL	CAN'T TELL

Naszay et al. (2018)	YES	YES	YES
	NO	NO	NO
	CAN'T TELL	CAN'T TELL	CAN'T TELL
Seifert et al. (2017)	YES	YES	YES
	NO	No	NO
	CAN'T TELL	CAN'T TELL	CAN'T TELL
Mackert et al. (2016)	YES	YES	YES
	NO	No	NO
	CAN'T TELL	CAN'T TELL	CAN'T TELL

1. QUALITATIVE STUDIES	1.4. - Is the interpretation of results sufficiently substantiated by data?	1.5. - Is there coherence between qualitative data sources, collection, analysis and interpretation?
Cabrita et al. (2019)	YES	YES
	NO	NO
	CAN'T TELL / Somewhat	CAN'T TELL / Somewhat
**2. QUANTITATIVE STUDIES**	**2.4. - Is the risk of nonresponse bias low?**	**2.5. - Is the statistical analysis appropriate to answer the research question?**
Bol et al. (2018)	YES	YES
	NO	NO
	CAN'T TELL	CAN'T TELL

König et al. (2018)	YES	YES
	NO	NO
	CAN'T TELL	CAN'T TELL
Naszay et al. (2018)	YES	YES
	NO	NO
	CAN'T TELL	CAN'T TELL
Seifert et al. (2017)	YES	YES
	NO	NO
	CAN'T TELL	CAN'T TELL
Mackert et al. (2016)	YES	YES
	NO	NO
	CAN'T TELL	CAN'T TELL
